# The Regulatory-associated protein of target of rapamycin 1B (RAPTOR 1B) interconnects with the photoperiod pathway to promote flowering in *Arabidopsis*

**DOI:** 10.1073/pnas.2405536122

**Published:** 2025-02-03

**Authors:** Reynel Urrea-Castellanos, Maria J. Calderan-Rodrigues, Anthony Artins, Magdalena Musialak-Lange, Appanna Macharanda-Ganesh, Alisdair R. Fernie, Vanessa Wahl, Camila Caldana

**Affiliations:** ^a^Max-Planck Institut für Molekulare Pflanzenphysiologie, Potsdam-Golm 14476, Germany; ^b^The James Hutton Institute, Dundee DD2 5DA, United Kingdom

**Keywords:** RAPTOR1B, TOR pathway, flowering, photoperiod, GIGANTEA

## Abstract

The transition from vegetative to reproductive growth, or floral transition, is a tightly regulated, energy-demanding process. In *Arabidopsis*, the interplay of light perception and circadian rhythms detects changes in photoperiod length, accelerating flowering under long days (LD). CONSTANS (CO), a transcription factor, upregulates FLOWERING LOCUS T (FT) in leaves during dusk. The FT protein then moves to the shoot apical meristem, triggering the floral transition. While light and circadian signals control CO protein levels, less is known about how the nutrients/energy sensing regulates the photoperiod pathway for flowering modulation in this process. In our study, we identify the contribution of the Regulatory-associated protein of target of rapamycin 1B (RAPTOR1B), a component of the nutrient-sensing TOR complex (TORC), in the induction of specific flowering genes under CO control. While transcription of CO remains unaffected in *raptor1b* mutants, a reduction in its protein levels at dusk is observed compared to the wild type. Remarkably, the mutant also exhibits compromised GIGANTEA (GI) protein levels, crucial for CO stabilization during dusk. Our results indicate that the interaction and colocalization of RAPTOR1B with GI in the nucleus might influence GI levels through an unknown posttranscriptional mechanism. Genetic crosses position RAPTOR1B upstream of CO and GI. This is supported by phenotypic and molecular analyses. Our findings demonstrate that RAPTOR1B, likely as part of TORC, contributes to the photoperiod pathway of the flowering network, ensuring the timely initiation of floral transition under LD conditions.

## Significance

For annual plants such as *Arabidopsis*, the correct flowering time is critical for reproductive success. While molecular mechanisms enabling plants to integrate day length with internal rhythms to accelerate flowering are well described, pathways sensing and conveying resource availability remain poorly understood. A potential player is the TARGET OF RAPAMYCIN (TOR) kinase, a master regulator of growth and nutrient sensing conserved in all eukaryotes. We found that the Regulatory-associated protein of target of rapamycin 1B (RAPTOR 1B), post-transcriptionally regulates a component of the photoperiod pathway of the flowering network, CONSTANS, via GIGANTEA. As such our study is the first to demonstrate an evolutionarily conserved connection between RAPTOR1B and the regulation of reproduction via photoperiod sensing in plants.

Plants are exposed to a ~24 h diel cycle of light and dark periods, which change in their length according to the season and latitude. For annual plants such as *Arabidopsis thaliana* (*Arabidopsis*) sensing the photoperiod is crucial to accelerate flowering when the days are getting longer in spring (1, 2). This timing of plant developmental transitions significantly impacts the production of offspring, aiming to grow and reproduce when the environmental conditions are favorable. Photoperiod-mediated flowering relies on the expression of the transcription factor CONSTANS (CO) in leaves, which in turn upregulates *FLOWERING LOCUS* (*FT*) (3, 4). FT is the florigen signal that moves from the leaves to the shoot apical meristem (SAM) to initiate floral transition (5). Accumulation of CO under long days (LD) results from an “external coincidence” mechanism that involves the circadian clock and photoperiod sensing (6). In short days (SD), clock-regulated *CO* gene expression and protein synthesis occur mainly after dusk, hindering CO accumulation since this protein is degraded in the dark (6, 7). In contrast, in LD, high gene expression of *CO* coincides with the light period, where blue and far-red light photoreceptors further stabilize CO protein at dusk (7, 8). Additionally, the circadian-associated protein GIGANTEA (GI) mediates the degradation of *CO* expression repressors, such as CYCLING DOF FACTOR 1 (CDF1) (9), while it positively regulates diel protein turnover of CO via the interaction with FLAVIN-BINDING KELCH REPEAT F-BOX 1 (FKF1) and the circadian photoreceptor ZEITLUPE (ZTL) (10). Thus, the FT-CO-GI module integrates circadian rhythms with light signals to accelerate flowering in LD (4).

The length of the day is directly related to the duration of photosynthesis. In *Arabidopsis*, carbon assimilation in the form of sugars (e.g., sucrose) can be stored as starch or readily used as a source of carbon to sustain growth and development (11, 12). Carbon availability plays a crucial role in restricting plant growth and development, with higher availability observed during longer photoperiods. A recent work has demonstrated that the regulation of photoperiodic growth—defined by the duration of photosynthesis in relation to day length—operates through a distinct molecular mechanism separated from that controlling photoperiodic flowering (13). Interestingly, disruption of the sugar sensing mechanism mediated by trehalose 6-phosphate (T6P) not only has an impact on the metabolic status of the plant, but also on the onset of flowering via the photoperiod pathway of the flowering network (14). This suggests the existence of players that are associated with both metabolic-driven growth and developmental transitions.

Another potential player integrating energy signaling into the flowering network is the TARGET OF RAPAMYCIN (TOR) kinase, a master regulator of growth and nutrient sensing conserved in all eukaryotes (15). In plants, REGULATORY ASSOCIATED PROTEIN of TARGET OF RAPAMYCIN (RAPTOR) is part of the target of rapamycin complex (TORC) together with TOR kinase and LETHAL with SEC13 PROTEIN 8 (LST8) (15). RAPTOR functions as the substrate recruiter for TOR, and its posttranslational regulation through phosphorylation mediates the regulation of TOR kinase activity (16, 17), indicating that RAPTOR can integrate specific signals to mediate TOR kinase substrates and/or its activity selectively. Although RAPTOR is widely recognized as a component of the TORC, it remains uncertain whether it additionally functions independently of TORC. RAPTOR is encoded by two homologs, *RAPTOR1A* and *RAPTOR1B*; however, only knockout mutants for the later gene exhibit a delay in developmental transitions, including flowering (18, 19). TORC integrates carbon availability information with external stimuli, such as light (20), which leads to the activation of various connected metabolic processes that steer growth and development (21). In *Arabidopsis*, chemical inhibition of TOR activity or downregulation of *TOR* expression restricts overall growth (22–26), while knocking out *TOR*, or its kinase domain, completely leads to embryo lethality (27, 28). Although mutants of *LST8-1* and *RAPTOR1B* display various developmental defects, they are viable and have been used to investigate the role of TORC (19, 29, 30).

Due to the pleiotropic effects of TORC malfunction at the seedling stage (21), it remains unclear whether the late flowering associated with TORC inhibition or mutations in *RAPTOR* and *LST8* genes (18, 19, 29) is solely a consequence of early developmental delays. To strictly investigate the role of TORC during flowering, we employed mutants of the *RAPTOR1B* gene, as their growth pattern closely mirrors that of wild-type plants under SD until the acquisition of floral competence. We found that RAPTOR1B promotes flowering under LD by regulating components of the photoperiod pathway of the flowering network. RAPTOR1B interacts with and promotes GI accumulation, thus contributing to CO abundance at dusk, which in turn, mediates the upregulation of *FT* and other downstream genes required for the floral transition. Our results demonstrate that RAPTOR1B feeds into the photoperiod pathway to ensure timely onset of flowering under LD.

## Results

### RAPTOR1B Positively Regulates Flowering.

**Fig. 1.**
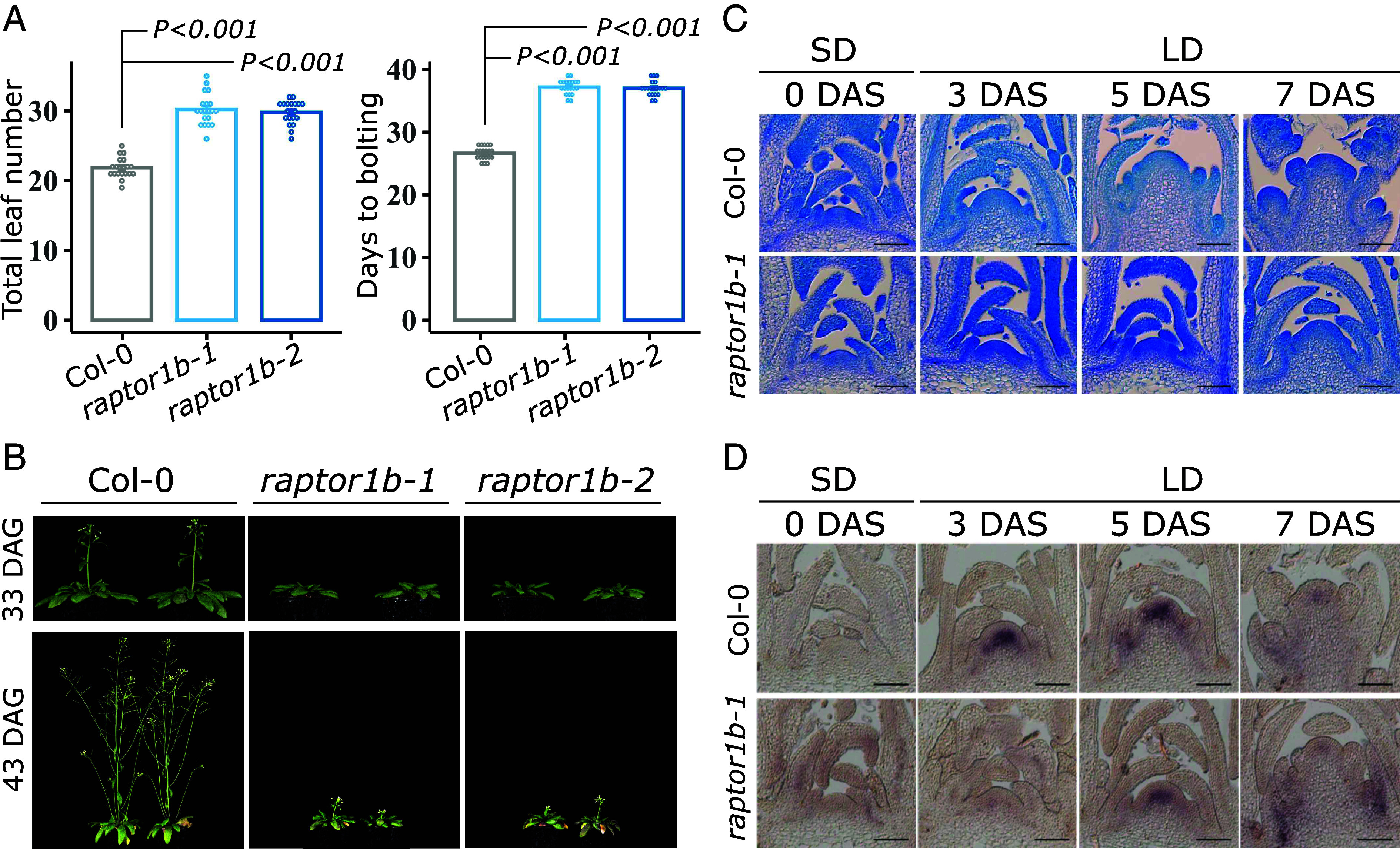
RAPTOR1B, the regulatory protein of the TOR complex, directly promotes flowering in *Arabidopsis* under LD conditions. (*A*) Total leaf numbers and days to bolting and total for Col-0 and two independent *raptor1b* mutant alleles, *raptor1b-1* and *raptor1b-2*, grown under LD conditions. Significant differences between the genotypes were determined by two-tailed Student’s *t* test (n = 20). Respective *P* values are provided. Error bars denote SE. (*B*) Representative images of plants used to determine flowering time in (*A*). Pictures were taken 33 and 43 days after germination (DAG). (*C*) Longitudinal sections through Col-0 and *raptor1b-1* apices stained with Toluidine blue. Plants were first grown in SD for 30 d and then shifted to LD to induce flowering. Apices were harvested before (0 DAS) and after the shift (3, 5, and 7 DAS). Apices were collected 1 h before dusk in SD and LD, respectively. DAS: days after shift. (Scale bar: 100 µm.) (*D*) RNA in situ hybridization using a specific probe for *SOC1* on Col-0 and *raptor1b-1* apices, for which plants were grown and harvested as in (*C*). DAS: days after shift. (Scale bar: 100 µm.)

To understand the impact of the *RAPTOR1B* mutation during the floral transition under LD conditions, two T-DNA insertion lines, named *raptor1b-1* and *raptor1b-2* (SI Appendix, Fig. S1*A*), were investigated. As previously reported (18, 19), both mutants bolted around 11 d later than wild type (Col-0). In addition, we observed an average of 8 extra leaves in the mutants under our growth conditions (Fig. 1 *A* and *B*). In contrast, the knock-out mutant for *RAPTOR1A, raptor1a-1,* did not display any visible changes (SI Appendix, Fig. S1 *B*–D) as previously described (18). Transformation of *raptor1b-1* plants with the *ProRAPTOR1B::6XMyc-RAPTOR1B or ProRAPTOR1B::RAPTOR1B-6XMyc* constructs were able to fully restore the total leaf number and recovered the days of bolting from 87.8% to 100% (SI Appendix, Fig. S2 *A* and B).

To exclude the possibility that the observed late flowering phenotype is a result of an overall developmental delay (18, 19), we initially cultivated plants first under SD conditions for 30 d. During this period, no obvious differences in the developmental stages were observed between wild-type and *raptor1b-1* plants, as supported by an equal number of rosette leaves at the end of the growth period (SI Appendix, Fig. S3 *A* and B). Subsequently, plants were transferred to LD to induce flowering. Wild-type plants showed an increase in SAM size upon floral transition and formation of flower primordia at day 3 and 5 after the shift, respectively. In contrast, in *raptor1b-1*, floral transition occurred much later, and no flower primordia were observed within the investigated time series (Fig. 1*C*). The later induction and attenuated expression in the apices of *SUPPRESSOR OF OVEREXPRESSION CO1* (*SOC1*) in *raptor1b-1* compared to wild type after the shift to LD supported the finding in the mutant (Fig. 1*D*). Consequently, later development of flower primordia in the mutant resulted in delayed bolting after the shift to LD (SI Appendix, Fig. S3 *C* and D). Furthermore, the floral transition and *SOC1* expression at the SAM were delayed in continuous LD conditions, even when the mutant seeds were sown 2 d in advance (staged *raptor1b-1*) to compensate for its later germination (SI Appendix, Fig. S4 *A* and B) (30). Altogether, these results suggest that the late flowering phenotype of *raptor1b-1* is not due to delays in an earlier developmental stage and that RAPTOR1B plays a role in the regulation of flowering.

### RAPTOR1B Promotes the Expression of Specific Flowering Genes.

**Fig. 2.**
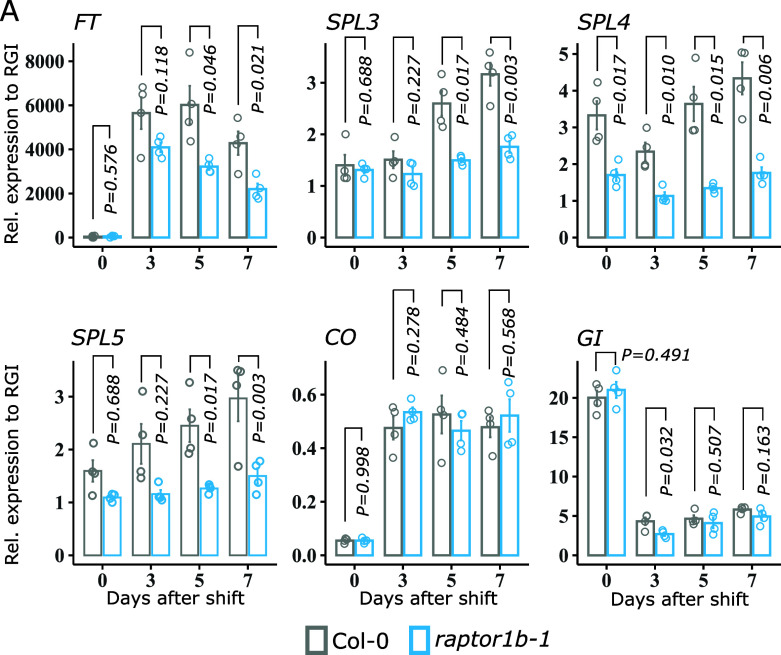
RAPTOR1B promotes the expression of flowering genes under LD conditions. (*A*) Gene expression analysis of *SPL3*, *SPL4*, *SPL5*, *FT*, *CO*, and *GI* by RT-qPCR (see SI Appendix, *Material and Methods* for detailed information about calculation of the relative expression). Col-0 and *raptor1b-1* plants were grown initially in SD for 30 d and then transferred to LD. Rosettes were harvested before (0 DAS) and after the photoperiod shift (3, 5, and 7 DAS). Plant material was collected as described in Fig. 1*C*. Significant differences between the genotypes for each day were determined by two-tailed Student’s *t* test (n = 4). Respective *P* values are provided. Error bars denote SE. DAS: days after shift.

We next measured the expression of key flowering genes in leaves of wild-type and *raptor1b-1* plants subjected to a photoperiod shift from SD to LD. As expected, this transfer induced *FT* expression in both genotypes (Fig. 2*A*). In *raptor1b-1*, however, *FT* levels were generally reduced, particularly at 5 and 7 d after the shift. Similarly, the closest homolog of *FT*, *TWISTER OF FT* (*TSF*) was induced under LD in both genotypes, but its expression in the mutant was affected in all time points (SI Appendix, Fig. S5*A*). The expression of *SQUAMOSA-PROMOTER BINDING PROTEIN-LIKE* (*SPL*) genes, in particular *SPL3, SPL4, and SPL5*, known to be induced in the rosette leaves and shoot apex upon induction to flowering (31), were likewise hampered in *raptor1b-1* upon transfer to LD (Fig. 2*A*). In contrast, other SPLs, such as *SPL9* and *SPL15,* were only significantly downregulated at day 7 in *raptor1b-1* compared to wild type (SI Appendix, Fig. S5*A*). A similar response in gene expression was observed for the second mutant allele *raptor1b-2* when subjected to the photoperiod shift (SI Appendix, Fig. S6*A*). We also monitored the expression of these marker genes in Col-0 plants transitioning from SD to LD after the treatment with the chemical inhibitor of TORC, AZD-8055. Similar to both *raptor1* alleles, we observed a reduction in the expression of *FT*, *TSF*, *FUL*, *SPL4*, and *SPL5* following the AZD-8055 treatment. *SPL3*, however, remained unaffected 7 d following the shift, suggesting that RAPTOR1B and also TOR would act synergistically in this response (SI Appendix, Fig. S7). Reduced expression at dusk in *raptor1b-1* of *FT* and *SPL3, SPL4,* and *SPL*5, but not of *SPL9* or *SPL15*, was further confirmed over three consecutive diel cycles during the floral transition in continuous LD (SI Appendix, Fig. S8*A*). Expression levels of *TOR* did not seem to be impaired in both mutant alleles, while *TOR* and *RAPTOR1B* expression levels remained stable upon the photoperiod shift in Col-0 (SI Appendix, Figs. S5*A* and S6A). Taken together, *RAPTOR1B* is required to promote gene expression of positive regulators of flowering, namely *FT* and *SPLs* (*3*, *4,* and *5*) under LD conditions.

### RAPTOR1B Contributes to Increasing CO Levels at Dusk on LD.

Expression levels of *FT*, *SPL3, SPL4, SPL5*, and *SOC1* are ultimately promoted by CO (8, 32). In contrast to the situation in SD, CO protein accumulates at dusk in LD as a result of both induced gene expression and light-mediated stabilization (6, 33, 34). *CO* expression itself was not affected in *raptor1b-1* neither at dusk during the SD to LD photoperiod shift (Fig. 2*A*) nor throughout a 3-d time course under continuous LD (SI Appendix, Fig. S8*A*). Moreover, *CO* expression in the second mutant allele was not compromised (SI Appendix, Fig. S6*A*). In contrast, *FT* expression was significantly downregulated in the mutant, particularly at dusk in both experiments (Fig. 2*A* and SI Appendix, Fig. S8*A*). These results indicate that the reduced expression of flowering-promoting genes observed in the *RAPTOR1B* mutant is not a consequence of altered *CO* gene expression.

**Fig. 3.**
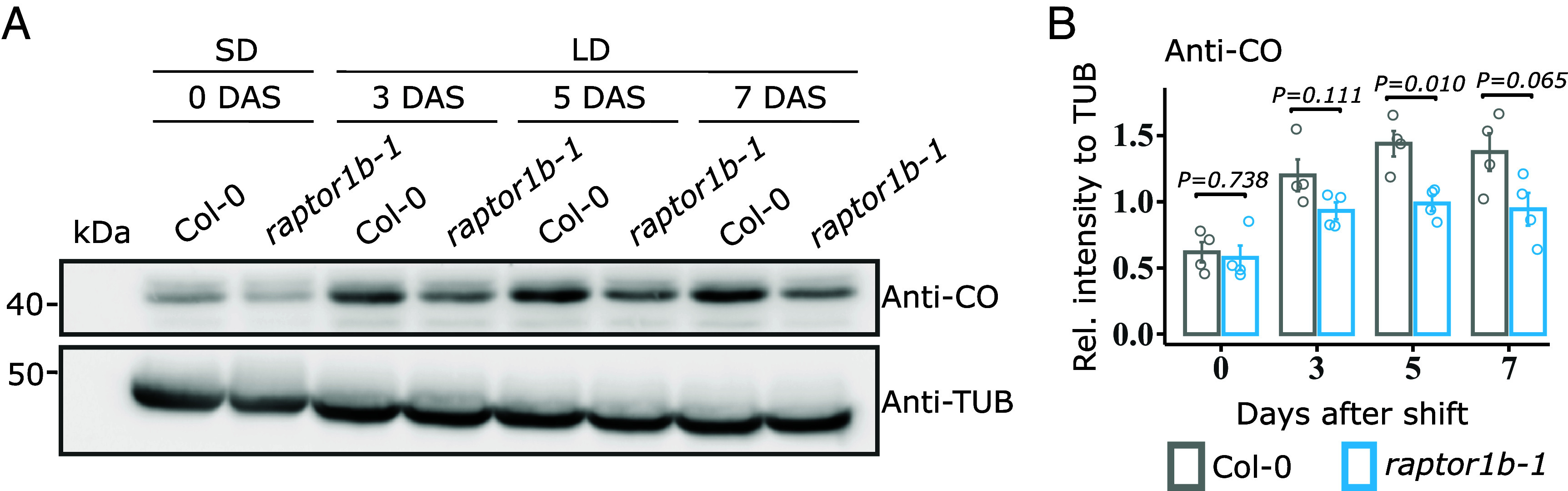
RAPTOR1B contributes to CO protein stability under LD. (*A*) Western blot analysis to determine protein levels of CO in Col-0 and *raptor1b-1* during a SD to LD shift experiment. Growth conditions and plant material are the same as described in Fig. 2*A*. Endogenous CO and TUBULIN proteins were immuno-detected using Anti-CO and Anti-TUB, respectively. One biological replicate is depicted here and the other three can be found in SI Appendix, Fig. S6*A*. (*B*) Relative CO protein levels calculated for the immunoblot in (*A*). Anti-CO was normalized to the respective Anti-TUB signal to determine the relative intensity at each day. Significant differences between the genotypes for each day were determined by two-tailed Student’s *t* test (n = 4). Respective *P* values are provided. Error bars denote SE.

Posttranscriptional regulation of components of the photoperiod pathway plays a major role in regulating flowering. As expected, LD triggered the accumulation of CO protein in both genotypes. However, protein abundance was reduced in *raptor1b-1* at all-time points upon shift to LD (Fig. 3 *A* and *B* and SI Appendix, Fig. S9*A*), suggesting that the absence of RAPTOR1B might affect CO protein accumulation or stability. To inspect the effect of the *raptor1b-1* mutation on CO regulation, we next applied the inhibitor of translation cycloheximide (CHX) and the proteasome inhibitor MG132 to *raptor1b-1* and wild-type seedlings undergoing the floral transition using a hydroponic system (SI Appendix, Fig. S9*B*). In wild type, CO levels increased at dusk from day 9 to 10 after germination when the plants were grown in continuous LD, while for *raptor1b-1*, CO accumulation was reduced compared to wild type. These observations resemble the differences in CO upregulation observed at the floral transition during the SD to LD shift experiment (Fig. 3 *A* and *B*). Although CHX treatment did not significantly alter CO levels at dusk for both genotypes compared to the mock treatment (DMSO), CO levels in *raptor1b-1* still displayed a reduced trend when compared to wild type. Remarkably, under proteasome inhibition (+MG132), CO levels increased in *raptor1b-1* compared to the mock, reaching similar levels to the MG132-treated wild-type plants (SI Appendix, Fig. S9*B*). The alterations in CO accumulation observed under MG132 treatment may suggest an enhanced degradation of CO protein in *raptor1b-1* at dusk.

### RAPTOR1B Interacts with and Regulates GI Protein Levels at Dusk.

**Fig. 4.**
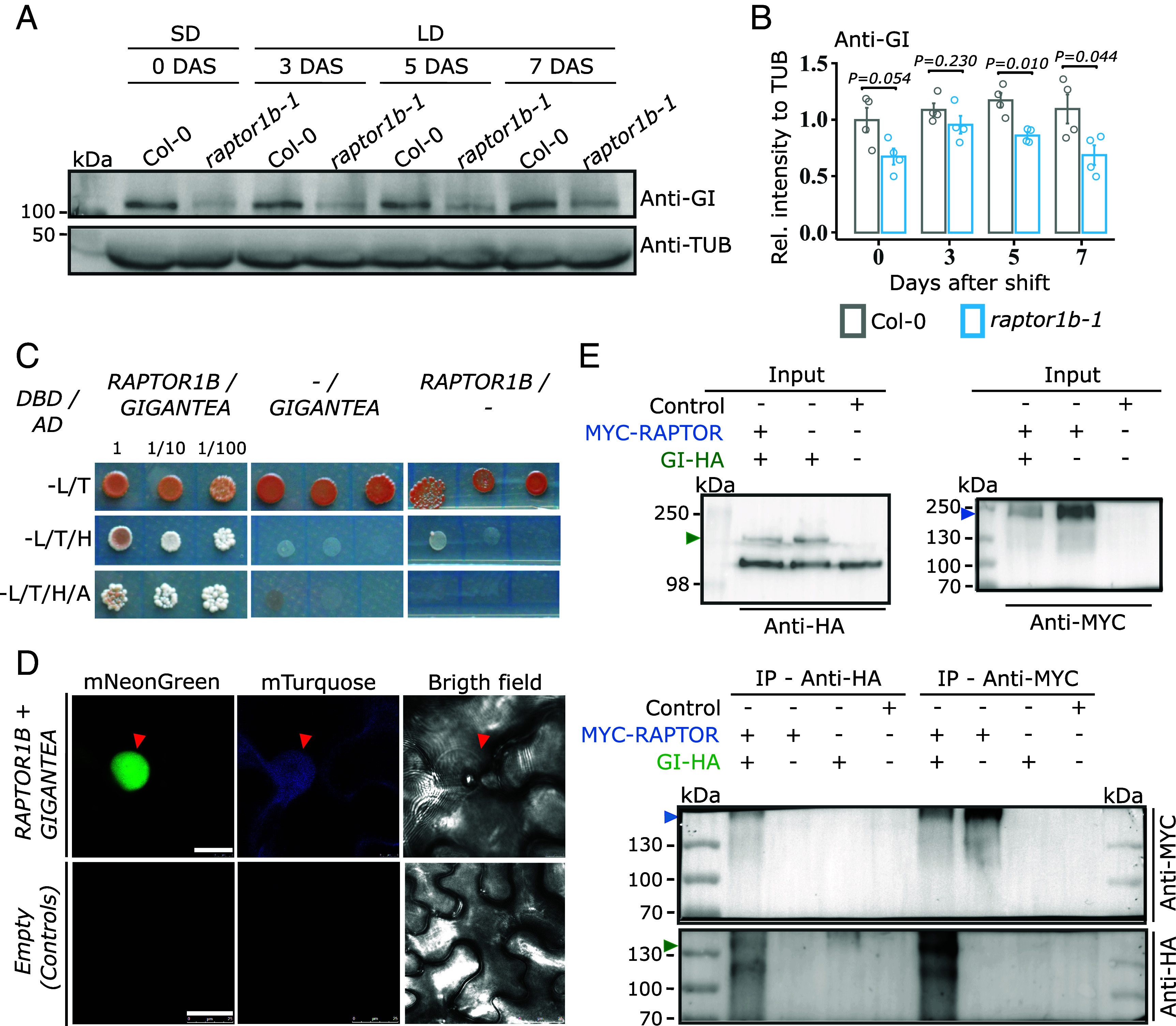
RAPTOR1B interacts with GI and promotes its stability at dusk. (*A*) Western blot analysis to determine protein levels of GI in Col-0 and *raptor1b-1* grown in a SD to LD shift regime (as described in Fig. 2*A*). Endogenous GI and TUBULIN proteins were immuno-detected using Anti-GI and Anti-TUB, respectively. One biological replicate is depicted here, three more can be found in SI Appendix, Fig. S7*C*. (*B*) Relative GI levels calculated for the immunoblot in (*A*). Anti-GI signal was normalized to the respective Anti-TUB signal to determine the relative intensity at each day. Significant differences between the genotypes for each day were determined by two-tailed Student’s *t* test (n = 4). Respective *P* values are provided. Error bars denote SE. (*C*) RAPTOR1B and GI interact in yeast. Full sequences of *RAPTOR1B* and *GI* were used. Yeast was spotted on synthetic double (-L/W), triple (-L/W/H), or quadruple (-L/W/H/A) dropout medium. RAPTOR1B and S6K1 interaction was included as positive control (SI Appendix, Fig. S8*A*). L (Leucine), W (Tryptophan), H (Histidine), A (Adenine). (*D*) Transient coexpression of *Pro35S::mTurquoise-RAPTOR1B* and *Pro35S::GIGANTEA-mNeonGreen* in *N. benthamiana* leaves. As control, the empty vector (*pMDC32-HPB*) was infiltrated. Red arrowhead indicates the nucleus. (Scale bars, *Upper* panel: 10 µm, *Bottom* panel: 25 µm.) Individual infiltration of both constructs was performed, respective images can be found in SI Appendix, Fig. S9 *A* and B. (*E*) Coimmunoprecipitation essays between RAPTOR1B and GI in planta. *Pro35S::6xMYC-RAPTOR1B* and *Pro35S::GI-3xHA* were transiently coexpressed in *N. benthamiana* leaves and protein extracts were immunoprecipitated (IP) with Anti-MYC or Anti-HA magnetic microbeads. Immunoblotting was performed using Anti-MYC and Anti-HA antibodies. As controls, *Pro35S::6xMYC-RAPTOR1B*, *Pro35S::GI-3xHA,* and an unrelated protein (FLZ14-mNeongreen) were expressed individually (Control). Protein extracts prior to the immunoprecipitation (Inputs) are depicted in the *Top* panels. Blue and green arrow heads indicate expected protein sizes for 6xMYC-RAPTOR1B (~160 kDa) and GI-3xHA (~132 kDa), respectively.

We next investigated whether the altered CO levels in *raptor1b-1* was associated with light signaling (4). Perception of blue light through CRYPTOCHROME 1 and 2 (CRY1 and CRY2) contributes to the abundance of CO at dusk by suppressing the ubiquitin degradation complex, which includes CONSTITUTIVE PHOTOMORPHOGENIC 1 (COP1) and SUPPRESSOR of PHYA-105 1 (SPA1) (35–37). Likewise, far-red light perception via PHYTOCHROME A (PHYA) stabilizes CO (7), probably through the inactivation of the COP1–SPAs complex (4). PHYA and CRY1 protein levels were similar at dusk under SD and LD in both genotypes (SI Appendix, Fig. S10 *A* and B), indicating no obvious connection between light sensing and the RAPTOR1B in regulating CO abundance. CO protein turnover is also regulated by a multilayered regulatory mechanism involving interaction among GI, FKF1, and ZTL (10). Given that GI acts by both stabilizing FKF1 and inhibiting ZTL to promote CO stability in the late afternoon, gene expression and protein levels of GI were assessed. *GI* transcript levels are unaffected in *raptor1b-1* (Fig. 2*A* and SI Appendix, Fig. S8*A*). In contrast, in both photoperiods, GI protein levels were reduced in the *raptor1b-1* mutant compared to the wild type (Fig. 4 *A* and *B* and SI Appendix, Fig. S10*C*). Similarly, AZD-8055 application during floral transition also reduces the GI protein levels (SI Appendix, Fig. S11). Altogether, these findings suggest that RAPTOR1B, possibly via the TOR pathway, might control CO abundance via GI function at the posttranscriptional level.

Interestingly, a yeast two-hybrid assay showed that RAPTOR1B interacted with GI and the 40S ribosomal protein S6 KINASE 1 (S6K1, positive control), but not with CO, FKF1, or ZTL (Fig. 4*C* and SI Appendix, Fig. S12 *A* and B). Confirming the RAPTOR1B interaction with GI in planta, coimmunoprecipitation assays using *Pro35S::6XMYC-RAPTOR1B* and *Pro35S::GI-3XHA* showed that both recombinant proteins pulled-down each other, when transiently coexpressed in *Nicotiana benthamiana* leaves (Fig. 4*E* and SI Appendix, Fig. S10*D*). To test for subcellular localization of this interaction, *Pro35S::mTurquose-RAPTOR1B* and *Pro35S::GI-mNeonGreen* were transiently coexpressed in *N. benthamiana*. Both reporters were shown to colocalize in the nucleus (Fig. 4*D*). In contrast to GI-mNeonGreen, mTurquose-RAPTOR1B signal was also observed in the cytoplasm and cytoplasmic strings (SI Appendix, Fig. S13 *A* and B). These results suggest that RAPTOR1B is involved in the photoperiod pathway through GI, increasing CO levels at dusk.

### GI and CO Act Downstream of RAPTOR1B.

**Fig. 5.**
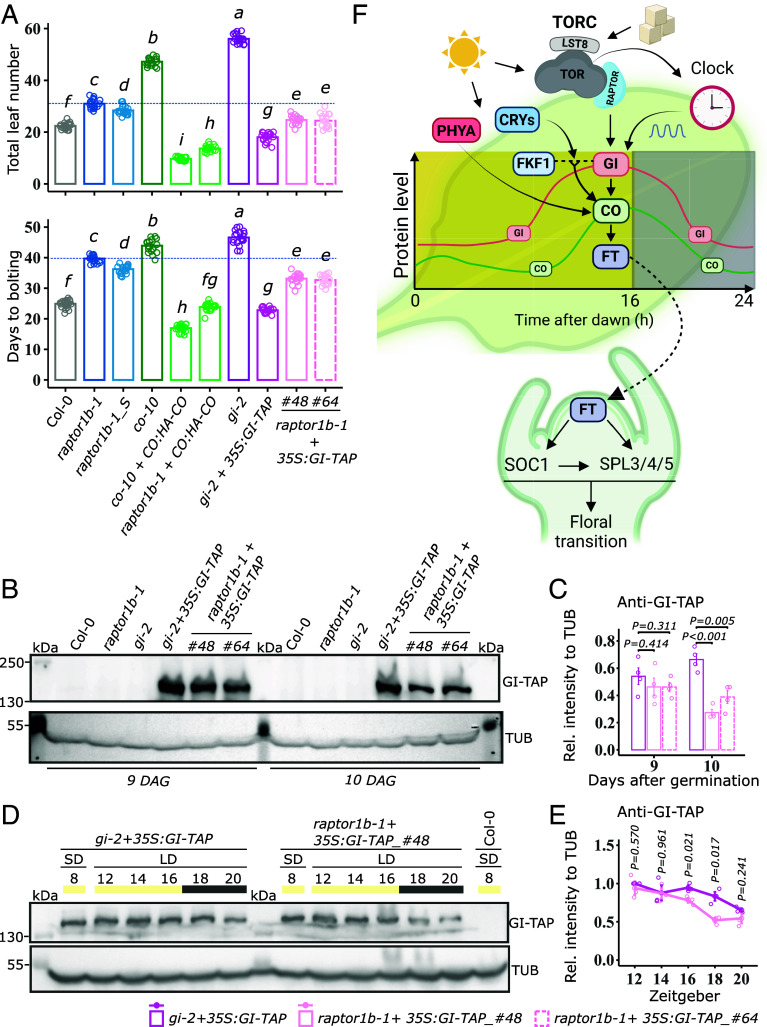
RAPTOR1B functions upstream of GI to contribute to CO stability at dusk and thus promote the floral transition in *Arabidopsis*. (*A*) Total leaf number and days to bolting under LD conditions for Col-0, *raptor1b-1*, *raptor1b-1_S* (staged), *co-10* (*constans* knock-out mutant), *co-10*+*ProCO:HA-CO* (complemented line for *co-10*), *raptor1b-1+ProCO:HA-CO* (*raptor1b-1* expressing HA-CO), *gi-2* (*gigantea* truncated mutant), *gi-2+Pro35S:GI-TAP* (complemented line for *gi-2*), and *raptor1b-1+Pro35S:GI-TAP* (*raptor1b-1* expressing GI-TAP, independent crosses: #48, #64). Significant differences among genotypes were determined by one-way ANOVA (*P* < 0.05) and a post hoc Tukey’s test as indicated by letters (n = 20). Error bars denote SE and a blue dotted line corresponds to the mean of *raptor1b-1*. (*B*) Western blot analysis of GI-TAP (~150 kDa). Plants were grown in a hydroponic system. Whole rosettes were harvested in the last hour before dusk at 9 and 10 DAG. GI-TAP was detected with Anti-GI and TUBULIN with Anti-TUB. One biological replicate is depicted here, three more can be found in SI Appendix, Fig. S11*C*. (*C*) Relative GI-TAP levels calculated for the immunoblot in (*B*). Anti-GI signal was normalized to the respective Anti-TUB signal for relative intensity. Significant differences between *gi-2+Pro35S:GI-TAP* and both *raptor1b-1+Pro35S:GI-TAP* lines (#48 an #64) for each day were determined by two-tailed Student’s *t* test (n = 4). Respective *P* values are provided. Error bars denote SE. (*D*) Western blot to determine GI-TAP abundance in *gi-2+35S:GI-TAP* and *raptor1b-1+Gi-TAP_#4*8 grown in a SD to LD shift regime. Rosettes were harvested at ZT8 in SD and every 2 h between ZT12 and ZT20 3 d after the shift in LD. Col-0 was included as control. Proteins were extracted and GI-TAP and TUBULIN were detected with Anti-GI and Anti-TUB, respectively. One biological replicate is depicted here and two more can be found in SI Appendix, Fig. S12*A*. (*E*) Relative GI-TAP levels calculated for the immunoblot in (*D*). Anti-GI signal was first normalized to the signal at 12 h in LD for each genotype and then to the respective Anti-TUB signal in the same running line. Significant differences between the genotypes for each time point were determined by two-tailed Student’s *t* test (n = 4). Respective *P* values are provided. Error bars denote SE. (*F*) Proposed model for the role of RAPTOR during floral transition in LD. The length of the light period is primarily sensed in leaves by the combined action of light signaling (mediated by PHYA and CRYs) and the circadian clock (driving gene expression of effector proteins, such GI and FKF1). This accounts for protein accumulation of CONSTANS at dusk, which in turn, upregulates the florigen, *FT*. FT protein travels from the leaves into the shoot apex via the vasculature to induce the expression of floral integrator genes (such SOC1) and flower promoting genes (such SPL3/4/5). GI, similar to CO, accumulates over the day and is degraded at night. Independent, or together with FKF1 in response to blue light-mediated by CRYs, GI helps to stabilize CONSTANS at dusk in LD. RAPTOR, which is part of the TORC, contributes to CO stability at dusk by promoting GI levels posttranscriptionally. TORC is known to be activated by light and sugars, suggesting that RAPTOR conveys nutrient status, in this case carbon availability, to regulate GI accumulation and thus fine-tune flowering via the photoperiod pathway. Remarkably, TOR is essential for sugar-mediated regulation of the circadian period, while GI integrates signals from the circadian clock, thus strengthening the potential crosstalk between these proteins.

We subsequently explored the genetic interplay between RAPTOR1B and the photoperiodic pathway of the flowering network. For this purpose, we performed crosses between *raptor1b-1* with previously characterized lines expressing either *Pro35S::GI-TAP* or *ProCO::HA-CO* (see SI Appendix for references) to augment the overall levels of these proteins within the mutant background. The ectopic expression of HA-CO in *raptor1b-1* mutant fully restored both bolting time and total leaf number to levels comparable to wild type (Fig. 5*A* and SI Appendix, Fig. S14*A*). However, this mutant (*raptor1b-1*+*ProCO::HA-CO*) still exhibited delayed flowering when directly compared to the transgenic background used for the cross (*co-10, ProCO::HA-CO*). Notably, this later transgenic line displayed the fastest bolting with the lowest total number of leaves among all tested genotypes. On the other hand, the partial recovery of both flowering time parameters was observed with the ectopic expression of GI-TAP in *raptor1b-1*, but it did not entirely recapitulate the Col-0 phenotype. Similar to the HA-CO scenario, *raptor1b-1* plants expressing GI-TAP still flowered later in comparison to the transgenic background employed for the cross (*gi-2+Pro35S::GI-TAP*). Subsequently, we examined the influence of *raptor1b-1* on the accumulation of GI-TAP and HA-CO in the crosses. Analysis of both chimeric proteins showed a reduction in their abundance at bolting at dusk (SI Appendix, Fig. S15 *A* and B), as previously observed for the endogenous proteins (Figs. 3*A* and 4*A*).

Finally, we investigated the impact of the *raptor1B-1* mutation on the protein turnover of GI-TAP before and after dusk (from ZT12 to ZT20) following a transition from SD to LD conditions. Under LD, the *raptor1b-1* mutation hindered GI-TAP accumulation, particularly at dusk and during the initial 4 h of the dark period as compared to the levels observed at ZT12 and ZT14 (Fig. 5 *D* and *E* and SI Appendix, Fig. S16*A*). These results suggest that in the presence of *raptor1b-1* mutation, the decay rate of GI-TAP is accelerated during the late afternoon and the early phase of the night. Confirming these observations, the abundance of GI-TAP at dusk was similarly dampened in the mutant background in plants undergoing the floral transition in a hydroponic system (Fig. 5 *B* and *C* and SI Appendix, Fig. S15*C*). In summary, our results indicate that ectopic expression of GI-TAP or HA-CO compensates for the diminished levels of the endogenous proteins caused by the lack of RAPTOR1B in the mutant. Conversely, when analyzed from another perspective, the introgression of *raptor1b-1* mutation into the background of the GI-TAP and HA-CO complementing lines diminishes their rescuing effect, indicating that GI and CO operate downstream of RAPTOR1B.

## Discussion

Photoperiod comprises complex interactions between light perception, sugar signaling, circadian rhythms, phytohormones, and temperature sensing allowing plants to adapt their developmental timing to seasonal changes. Here, we investigated the effect of the interplay between RAPTOR1B, a component of TORC, and the photoperiod pathway on the floral onset in *Arabidopsis*. A delayed flowering phenotype has been associated with the TOR pathway dysfunction (22), lack of RAPTOR (18, 19), or LST8 (29). By conducting independent experiments—one focusing on carefully staging *raptor1b-1* mutants through a developmental series and the other on transitioning them from SD to LD conditions—we observed a delayed shift from vegetative to inflorescence SAM, along with reduced *SOC1* expression, a key integrator of flowering signals and marker gene for reproductive success (38) (Fig. 1 *C* and *D* and SI Appendix, Fig. S4 *A* and B). Our results demonstrated that RAPTOR1B promotes the floral transition independently of other pleiotropic functions during earlier developmental stages, in which TORC also plays a role (21).

The reduction in *FT* expression under LD conditions and chemical inhibition of TORC suggest a disruption in the photoperiodic pathway of flowering network. This emphasizes the involvement of RAPTOR1B in the timing of floral transition via *FT* under LD conditions (32). CO conveys multiple signals to induce the expression of *FT* and eventually *SOC*1 in LD, promoting flowering (3, 4). We showed that lack of RAPTOR1B does not alter *CO* gene expression (Fig. 2*A* and SI Appendix, Fig. S8*A*) but instead its protein accumulation at dusk (Fig. 3 *A* and *B* and SI Appendix, Fig. S9*A*). Chemical inhibition of 26S proteasome-mediated degradation showed a recovery trend in CO levels in *raptor1b-1* (SI Appendix, Fig. S9*B*), suggesting that RAPTOR1B may play a role in maintaining CO stability at dusk.

Multiple mechanisms control the CO stability during flowering (7–9), including the phosphorylation status (39, 40). Our analysis revealed that CRY1/CRY2 and PHYA, crucial for light-mediated CO stability at dusk (7, 35–37), remained unaffected in *raptor1b-1* (SI Appendix, Fig. S10 *A* and B), weakening the hypothesis that light triggers the RAPTOR1B-mediated CO accumulation. GI integrates signals from circadian clock, day-length, and environmental cues such as light and temperature to tune developmental processes (41). GI along with ZTL and FKF1, transduces blue-light signals critical for perceiving the duration of light exposure during photoperiod, and flowering initiation (42, 43). In *raptor1b-1,* the protein abundance of GI, but not the oscillation of its transcript levels (SI Appendix, Fig. S8*A*) were reduced (Fig. 4 *A* and *B* and SI Appendix, Fig. S10*C*). Due to the GI influence on CO accumulation at dusk (10), our results pointed to a post-transcriptional misregulation of GI in the *raptor1b-1* mutant. We find that RAPTOR1B interacts with GI in vivo using ectopic overexpression in *N. benthamiana* (Fig. 4 *C* and *E*), but not with CO, FKF1, or ZTL (SI Appendix, Fig. S12 *A* and B). We were able to confirm the interaction between RAPTOR1B and GI using two independent methods in yeast [yeast two hybrid (Y2H), in vitro] and tobacco (co-IP, in vivo); however, it remains to be confirmed whether this interaction takes place in vivo using native promoters in *Arabidopsis*. However, as our current hypothesis is that the interaction is likely transient, this will be challenging to capture using currently available in vivo methods. In addition, it remains still unclear whether this interaction is direct or, as recently reported for the TORC (44), requires additional players. Detailed further studies will therefore be required to elucidate the precise nature of this interaction.

Similar to *raptor1b-1*, *gi* mutants exhibit delayed flowering primarily due to downregulation of CO and *FT* (45). Accordingly, ectopic expression of GI-TAP partially recovered the late flowering phenotype in the *raptor1b-1* background, while HA-CO expression fully restored the flowering time parameters (Fig. 5*A* and SI Appendix, Fig. S14*A*). The lack of capacity of the GI-TAP transgene to fully complement the phenotype of the *raptor1b-1* mutation might be explained by insufficiently high protein levels (Fig. 5 *B*–*E*). Taken together, this supports the idea that RAPTOR1B controls CO levels at dusk through GI function, positioning both components of the photoperiod pathway of the flowering network downstream of RAPTOR1B (Fig. 5*F*).

Given the emerging role of the TORC in protein stability (44), one possibility is that RAPTOR1B may recruit GI to be phosphorylated by the TOR kinase. This is a plausible explanation, given that two phosphosites, S178 and S169, were identified in GI phosphoproteome (46) and additional S and T residues can be potentially phosphorylated. Although the role of this posttranslational modification in GI-mediated floral induction remains to be explored. Moreover, the nuclear colocalization of RAPTOR1B and GI (Fig. 4*D* and SI Appendix, Fig. S13 *A* and B) provides additional support for their role in flowering, given that GI needs to be nuclear localized to exert its function (9, 45). It is possible that RAPTOR1B interacts with the middle portion of GI, responsible for its nuclear localization (47) where it activates flowering by binding the LOV domains of other floral inducers (48). Hence, the subcellular compartment of the interaction and the posttranscriptional modification of GI mediated by RAPTOR1B need to be investigated in the future.

The signals through which RAPTOR1B controls GI abundance during floral transition remain to be elucidated. An obvious signal might have been light; however, our data do not support this hypothesis. The *GI* expression oscillation following circadian control (49, 50) was not affected in *raptor1b-1* plants (SI Appendix, Fig. S8*A*), nor were the protein peaks shifted (Fig. 5*D*). The circadian regulation of GI stability involves E3 ubiquitin-ligase COP1 and the clock-associated proteins ELF3 (51) and it cannot be excluded that any of these factors play a role in the mechanism behind the RAPTOR 1B-dependent effect on GI here presented.

Another plausible mechanism may implicate carbon availability, since the stability of GI relies on sucrose levels (52). Mutations in *raptor1b* decrease sucrose content (30), which might contribute to the mediation of sucrose-dependent GI stability. To sustain high CO protein levels at dusk in LD, not only its high gene expression levels need to coincide with its light-dependent stabilization (partially mediated by GI), but also with the use of the energy surplus in form of sugars (e.g., sucrose) occurring at dusk and early night in LD (12, 53). In LD, the lower sucrose levels in the *raptor1* mutant is followed by higher starch content, suggesting a shift toward carbon storage, and consequently less available carbon to fuel growth (19). A conceivable scenario might integrate the function of RAPTOR1B with the “external coincidence model” occurring in long photoperiods to induce flowering (6). This hypothesis coincides with the observed misregulation of GI in *raptor1b* mutants occurring specifically at the end of the day and early hours of the dark period (Fig. 5 *D* and *E*). Recently, it was shown the photoperiodic regulation of photosynthetic growth and flowering can be uncoupled (13). In our work, we provided evidence that RAPTOR1B feeds into the photoperiod pathway of the flowering network. Considering the role of the TORC pathway in sensing the metabolic status, we cannot exclude a crosstalk with photoperiod growth-related processes. The T6P pathway is one of the players at the intersection of nutrient signaling, growth, and specific developmental routes, feeding sucrose signals into the floral transition downstream of the photoperiod pathway of the flowering network at the level of *FT* (14).

The photoperiod and TOR pathways are interconnected through circadian regulation (54), nutrient signaling (29, 55), temperature changes (56), and stress responses (57) across a variety of species. In plants, an emerging role of the TOR pathway in regulating photoperiod-dependent photosynthetic growth has been highlighted in previous studies (19, 29). During photosynthetic growth, evidence suggests that the components of the TOR pathway, as a major nutrient sensor, integrates light and circadian perception with specific carbon partitioning strategies that correspond to different photoperiods (58, 59). Our work demonstrated that RAPTOR1B interacts with the photoperiod pathway of the flowering network in plants. GI homologs, conserved across vascular plants, control photoperiodic flowering (60), while their absence in nonvascular plants suggests that plants evolved this mechanism during land colonization to adapt to changing environments and ensure reproductive success. Remarkably, longer photoperiods regulate the TOR pathway to advance reproductive stages in *Drosophila* (61), promote spermatogenesis in roosters (62), and influence the testicle maturation of hamsters (63), reinforcing the direct link of photoperiod, TORC, and reproductive success in different groups of organisms.

## Material and Methods

### Plant Material and Growth Conditions.

All *A. thaliana* plants used in this study are in the Columbia (Col-0) background (wild type). Reference of the mutants and description of the crosses used in this study are found in SI Appendix, *Material and Methods*. A detailed description of growth conditions for the flowering time and SD to LD photoperiod shift experiments, as well as for the chemical treatments with CHX (100 µM) and the proteasome inhibitor MG132 (100 µM) performed in the hydroponics system, can be found in SI Appendix, *Material and Methods*.

### Generation of Complementing Transgenic Lines in *A. thaliana*.

To complement the *raptor1b-1* mutant, 6XMyc was fused in-frame either at the N terminus or C-terminus of RAPTOR1B gene under the control of its endogenous promoter (*ProRAPTOR1B::6XMyc-RAPTOR1B*, *ProRAPTOR1B::RAPTOR1B-6XMyc*). Constructs were stably transformed by the floral dip method using *A. tumefaciens* strain GV3101. For details of plasmid construction and plant selection, refer to SI Appendix, *Material and Methods*.

### Y2H.

Complete coding sequences of *RAPTOR1B* (*AT3G08850*), *GIGANTEA* (*AT1G22770*), *CONSTANS* (*AT5G15840*), *FKF1* (*AT1G68050*), and *ZTL* (*AT5G57360*) were used to generate the required Y2H constructs. For *S6K1* (*AT3G08730*), a region spanning from amino acid 9 to 293 was employed. Detailed information about plasmid construction and the conditions for the Y2H assay are described in SI Appendix, *Material and Methods*.

### Gene Expression and RNA In Situ Analysis.

For qRT-PCR, total RNA was extracted from approximately 50 mg of finely minced tissue in all cases using the Quick-RNA™ Plant Miniprep kit from ZYMO RESEARCH® (ref. R2024). Further details regarding cDNA synthesis, expression analysis, and a list of primers used can be seen in SI Appendix, Table S1 and *Material and Methods*. The probe to detect *SOC1* expression at the SAM as well as the RNA in situ hybridization method are described in SI Appendix, *Material and Methods*.

### Protein Extraction and Immunoblot Analysis.

First, 50 mg of finely powdered tissue was suspended in 150 µL (3 volumes) of 2× extraction buffer [0.125 M Tris-HCl, pH 6.8; 4% SDS (v/v); 20% (v/v) glycerol; 0.01% (w/v) Bromophenol blue; 10% ß-mercaptoethanol], mixed vigorously, and incubated at 95 °C for 5 min. Denatured proteins were run in 8% (v/v) acrylamide gel containing 0.1% SDS and then transferred into a PDVF membrane. Additional details about primary and secondary antibodies, running conditions, blotting, detection, and analysis can be found in SI Appendix, *Material and Methods*.

### Coimmunoprecipitation of Proteins Expressed in *N. benthamiana*.

Full-coding sequences of RAPTOR1B (*AT3G08850*) and GIGANTEA (*AT1G22770*) were fused with 6XMyc and 3XHA to generate *Pro35S::6XMyc-RAPTOR1B* and *Pro35S::GI-3XHA*, respectively. Constructs were transiently coexpressed in leaves of 6-wk-old *N. benthamiana* by leaf infiltration with *A. tumefaciens* strain GV3101. Proteins were extracted from 1 g of finely minced tissue. Further details about protein extraction, coimmunoprecipitation, and antibodies are found in SI Appendix, *Material and Methods*.

### Protein Localization by Transient Expression in *N. benthamiana*.

Six-week-old *N. benthamiana* leaves were infiltrated with *A. tumefaciens* strain GV3101 harboring *Pro35S::mTurquose-RAPTOR1B* or *Pro35S::GI-mNeonGreen.* Leaf pieces were visualized 48 h after infiltration using a SP5 confocal laser scanning microscope (©Leica Microsystems). Details about image acquisition and constructs are in SI Appendix, *Material and Methods*.

### Data Visualization and Statistical Test.

RStudio version 1.4.1717 was used for statistical analysis and plotting (https://www.rstudio.com). Further details are provided in SI Appendix, *Material and Methods*.

## Supplementary Material

Appendix 01 (PDF)

## Data Availability

All study data are included in the article and/or SI Appendix.
