# Supplementary material for: The Regulatory-associated protein of target of rapamycin 1B (RAPTOR 1B) interconnects with the photoperiod pathway to promote flowering in Arabidopsis
Source: Proc Natl Acad Sci U S A. 2025 Feb 3;122(6):e2405536122. doi: 10.1073/pnas.2405536122 (PMC11831161; doi:10.1073/pnas.2405536122)
Supplement: Supplementary file 1 — Appendix 01 (PDF) [file pnas.2405536122.sapp.pdf]

Supporting Information for

The Regulatory-Associated Protein of TOR 1B (RAPTOR1B) interconnects with the photoperiod pathway to promote flowering in *Arabidopsis*

Reynel Urrea-Castellanos[a], Maria J. Calderan-Rodrigues[a,1], Anthony Artins[a], Magdalena Musialak-Lange[a], Appanna Macharanda-Ganesh[a], Alisdair R. Fernie[a], Vanessa Wahl[a,b], Camila Caldana[a,2]

[a]Max-Planck Institut für Molekulare Pflanzenphysiologie, 14476, Potsdam-Golm, Germany

[b]The James Hutton Institute, DD2 5DA, Dundee, United Kingdom

[1] Present address: Universidade de São Paulo, Escola Superior de Agricultura "Luiz de Queiroz", 13418-900, Piracicaba, SP, Brazil. [2] To whom correspondence may be addressed. Email: caldana@mpimp-golm.mpg.de

This PDF file includes:

Supplementary text
Figures S1 to S16
Table S1
SI References

Supporting Information Text

Material and Methods

Plant material and growth conditions

All *Arabidopsis thaliana* plants used in this study are in the Columbia (Col-0) background. The mutant alleles for *RAPTOR1B* gene (*AT3G08850*), *raptor1b-1* (SALK_101990) and *raptor1b-2* (SALK_022096), were obtained from NASC and previously described (1, 2). *RAPTOR1A* gene (*AT5G01770*) mutant allele *raptor1a-1* (SALK_043920) was ordered from NASC and described before (1, 3, 4). *GIGANTEA* (*GI*) mutant *gi-2* and *GIGANTEA* complementing line *gi-2+Pro35S::GI-TAP* were provided by Prof. Alex Webb (University of Cambridge, UK) and described previously (5–8). *CONSTANS* (*CO*) mutant *co-10* (SAIL_24_H04) and complementing line *co-10+ProCO::HA-CO* were previously described (9, 10). *gi-2+Pro35S::GI-TAP* and *co-10+ProCO::HA-CO* were crossed with *raptor1b-1* (as pollen donor) and selected for homozygous *raptor1b-1* and presence of Pro*35S::GI-TAP* and *ProCO::HA-CO* constructs.

For flowering time experiments, seeds were first sown in a 1:1 mixture of soil (Stender) with vermiculite, stratified at 4°C for 3 days in darkness, and then transferred into a growth chamber. Plants were grown under a long photoperiod (LD, 16 h light / 8 h dark) with light intensity of 150 µmolm^{-2}s^{-1} and temperatures of 22°C / 18°C (light / dark). Bolting time was recorded when the inflorescence stem reached ≥ 0.5 cm. At this stage, total leaf number was determined after counting rosette and cauline leaves. For the short day (SD, 8 h light / 16 h dark) to long day (LD, 16 h light / 8 h dark) shift experiments, light intensity and temperature were kept at 160 µmolm^{-2}s^{-1} and at 22°C (day and night, to exclude the influence of temperature), respectively. Rosettes were harvested in the growth chambers and rapidly snap frozen in liquid nitrogen. Number of harvested individuals per replicate and time of the harvest are indicated in the figure legend of each experiment.

Hydroponic *in-vitro* experiment with MG132, Cycloheximide (CHX) and AZD-8055 treatments

For the *in vitro* experiment with the proteasome, translational inhibitors, and the chemical TOR inhibitor AZD-8055 Col-0 and *raptor1b-1* plants were grown for 10 days in a hydroponic system following the protocol described in (11). At this stage, plants are going through the floral transition. Growth conditions: LD (16 h light / 8 h dark) with temperatures of 21°C (day) and 19°C (night), light fluence rate 160 µmolm^{-2}s^{-1}, and HR of 60%. 10 days after germination, 100µM cycloheximide (Sigma-Aldrich, cat no. C7698), 100µM MG132 (Sigma-Aldrich, cat no. 4747908), 2µM AZD-8055 (MedChemExpress, cat no. HY-10422) or DMSO (1 % v/v) (mock control) were added to the media 30 minutes before dawn. Next, shoots were harvested 15 h after dawn, either before or after the treatment on days 9 and 10, respectively. For the TOR inhibited plants, Col-0 shoots were harvest only after the treatment. Samples were snaped-frozen in liquid nitrogen and kept at –80°C for further analysis.

AZD treatment of plants grown in soil during the SD to LD photoperiod shift

To evaluate the effect of inhibiting TOR on the expression of flowering genes during the floral transition, Col-0 was grown for 30 days in SD conditions and then transferred to LD as described above. AZD-8055 (10 µM, MedChemExpress, cat no. HY-10422) and DMSO (1 % v/v, mock control) were sprayed on rosette leaves 30 min before the light was switched on at days 0, 2, 4, and 6 after the photoperiod shift. As a control, plants were also harvested without any treatment on day 0 (“None” in the plots). For both treatments, individual plants were sprayed 6 times at the day of treatment. To extract RNA, whole rosettes were harvested 1 h before dusk at days 0, 3, 5, and 7 after the photoperiod shift. RNA extraction was performed as described below.

Generation of complementing transgenic lines in *Arabidopsis thaliana*

PCR amplification from DNA or RNA templates was carried out using Phusion™ High-Fidelity DNA Polymerase (Thermo Scientific, ref. F-530XL). A list of primers is provided in Table S1. *ProRAPTOR1B::RAPTOR1B-6xMyc* construct was generated by amplifying first the coding sequence of *RAPTOR1B* (*$RAPTOR1B_{cds}$,* lacking the stop codon) from *A. thaliana* (Col-0) cDNA and cloned via *In-Fusion*® (Takara Bio, ref 638948) in frame into the *pE3c* vector before the *6XMyc* tag (Addgene, https://www.addgene.org). Next, the promoter region of *RAPTOR1B* (1363 bp fragment upstream from the start codon) was amplified from genomic DNA and cloned via *In-Fusion*® cloning upstream of *$RAPTOR1B_{cds}$-6xMyc* in the pE3c plasmid to generate *ProRAPTOR1B::RAPTOR1B-6xMy.* Subsequently, via Gateway cloning (Invitrogene™), *ProRAPTOR1B::RAPTOR1B-6xMy* was subcloned into the plant expression vector *pGWB501* (Addgene, https://www.addgene.org), which harbors the

Hygromycin resistance gene for plant selection. For *ProRAPTOR1B::6xMyc-RAPTOR1B* construct, *RAPTOR1B*$_{cds}$ was amplified conserving the stop codon and cloned in frame into the *pE3n* vector downstream of the 6XMyc tag (Addgene, https://www.addgene.org). Subsequently, cloning the promoter region and subcloning into the plant destination vector, *pGWB501*, was done as described above. Both constructs were transformed into the *raptor1b-1* mutant background by the floral dip method (12) using *A. tumefaciens* (strain GV3101). Selection of positive transformants and homozygous transgenic lines in the F3 was performed in ½ MES media containing Hygromycin as described in (13). At least three independent transgenic lines for each construct were chosen for further analysis.

Yeast two hybrid

Full coding sequences of all genes were first cloned into the entry vector *pDONR221*^{TM} using Gateway cloning (Invitrogene^{TM}). Next, *RAPTOR1B* was recombined into *pDEST*^{TM}*32* (Invitrogene^{TM}), which harbors the DNA Binding Domain (DBD) at the N-terminal. *GIGANTEA*, *CONSTANS, FKF1, ZTL* and *S6K1* were recombined into *pDEST*^{TM}*22* (Invitrogene^{TM}), which harbors the Activation Domain (AD) at the N-terminal. The yeast strain Y2HGold (Takara Bio, ref. 630498) was double transformed with the bait plasmid (*DBD-RAPTOR1B*) in combination with each of the prey plasmids (*AD-GIGANTEA*, *AD-CONSTANS*, *AD-FKF1*, *AD-FKF1* and *AD-S6K1*) using the protocol in (14). As negative controls, empty *pDEST*^{TM}*32* and *pDEST*^{TM}*22* were cotransformed with the prey and bait plasmids, respectively. Positive transformants were selected in double dropout media lacking Leucine and Tryptophan (- L/W) (Taka Bio, ref. 630495). Subsequently, to test the interaction between *RAPTOR1B* and the prey proteins, recovered colonies from the previous selection were resuspended in 1X TE buffer and spotted into double (-L/W), triple (- L/W/H), and quadruple (- L/W/H/A) dropout media, as previously described (14). Primers are listed in Table S1.

RNA extraction and gene expression analysis

Total RNA was extracted from approximately 50 mg of finely grounded tissue using the Quick-RNA^{TM} Plant Miniprep kit from ZYMO RESEARCH® (ref. R2024). 1.8 -2 µg of total RNA was used for cDNA synthesis using the RevertAid H Minus First Strand cDNA Synthesis Kit from ThermoFisher (ref. K1632) and Oligo(dT)$_{18}$ as primer. 1 to 10 dilution of the synthesized cDNA was employed for further analyses. For gene expression analysis, qRT-PCR was carried out utilizing Power SYBR Green PCR-Master_Mix (Applied Biosystems, ref. 4367659) and the ABI PRISM 7900HT system (Applied Biosystems) for detection. Selection of the two reference genes (either *AT1G13320, AT4G34270, AT4G27960 or AT4G26410*) was performed for each experimental setup as described in (15), and the geometric mean of two selected reference genes (RGI) was used for further normalization. The comparative cycle threshold (CT) method was employed to determine the relative expression of the selected genes (16). In short, relative expression of the target gene was first normalized to the RGI to generate the ΔCt, and then normalized to the maximum value (referred as Max_Calibrator) to calculate the ΔΔCt. Next, final expression was computed employing the $2^{-\Delta\Delta Ct}$ formula. Four biological replicates per time point and genotype were performed by harvesting a pool of ~5 plants in each case. Significant differences were determined by Student's *t-test*. Information on the primers can be found in Table S1.

RNA *in situ* hybridization

The probe to detect *SOC1* expression at the SAM, as well as the RNA in situ hybridization method, were previously described in (17, 18).

Protein extraction and immunoblot analysis

50 mg of finely powdered tissue was suspended in 150 µl (3 volumes) of 2X extraction buffer (0.125 M Tris-HCl, pH 6.8; 4% SDS (v/v); 20% (v/v) glycerol; 0.01% (w/v) Bromophenol blue; 10% ß-mercaptoethanol), mixed vigorously and incubated at 95°C for 5 min. Subsequently, samples were centrifuged twice at 13,000 g for 5 min to remove cell debris. Denatured proteins were loaded and run into an 8% (v/v) Acrylamide gel containing 0.1% (v/v) SDS using a Bio-Rad Mini-PROTEAN Tetra System. Next, separated proteins were transferred into a 0.45 µm PDVF Immobilon-P membrane (Merck Millipore, ref. IPVH00010) and the PDVF membrane was blocked by incubation for 2 h at RT with 1X TBS-T buffer (20 mM Tris, 150 mM NaCl, pH 7.6, 1 $ml.L^{-1}$ Tween20) supplemented with 5% fat free milk. Primary antibodies, Anti-CONSTANS (PhytoAB, ref. PHY2297; 1:1000), Anti-GIGANTEA (Agrisera, ref. AS121864A; 1:1000), Anti-TUBULIN (Sigma-Aldrich, ref. T5168; 1:10000), Anti-CRY1 (PhytoAB, ref. PHY1707S; 1:1000) and anti-PHYA (PhytoAB, ref. 1907; 1:1000), were added and incubated with the membrane in 1X TBS-T buffer supplemented with 1% fat free milk overnight at 4°C. Following, after washing out the primary antibody 3 times with 1X TBS-T buffer, PDVF membrane was incubated with the secondary antibody, either Goat Anti-Rabbit (Bio-Rad, ref. 1706515) or Anti-Mouse (Bio-Rad, ref.

1706516) IgG (H + L)-HRP. Secondary antibodies were incubated in 1X TBS-T buffer containing 1% fat-free milk at a 1:3000 dilution for 2 h at RT. Immunoblots were imaged using a G:BOX Chemi XX6 system (Syngene) after adding SuperSignal West Femto reagents (Thermo Scientific, ref. 34095) directly to the PVDF membrane. Relative protein abundances were estimated with the help of Fiji software (19) as follows: each band's signal intensity was calculated and then normalized to the corresponding Anti-TUBULIN signal or to Ponceau staining in the same running line. As an exception, for Figures 5D and E, the immunoblot signal was first normalized to the respective signal at 12 h for each genotype and then to the respective Ani-TUBULIN in the corresponding running line (clarification included in the legend). At least three independent biological replicates for the same experiment were done for each immunoblot.

Coimmunoprecipitation of proteins expressed in *Nicotiana benthamiana*

To test the protein interaction between RAPTOR1B and GIGANTEA (GI) *in planta*, the complete coding sequences of both genes were amplified using *cDNA* prepared from *A. thaliana* Col-0 plants at the vegetative stage. For *Pro35S::6xMyc-RAPTOR1B* construct, previously cloned *6xMyc-RAPTOR1B* in *pE3n* for the complementation lines in *A. thaliana* (see above) *was* subcloned via Gateway into the plant destination vector *pMDC32-HPB* (Addgene, https://www.addgene.org), which harbors a 2X CaMV 35S promoter upstream of the insertion cassette to generate *Pro35S::6XMyc-RAPTOR1B*. For *GIGANTEA, Pro35S::GI-3XHA* construct was generated by cloning first into *pE2c* and subsequently into *pMDC32-HPB* as described for *RAPTOR1B*. Both constructs were transformed into *A. tumefaciens* (strain GV3101). For *N. benthamiana* leaf infiltration, *A. tumefaciens* harboring the constructs were coinfiltrated into 6-week-old plants as previously described (20). 48 h after the infiltration, leaves were cut and snap frozen into liquid nitrogen and stored at -80 Cº. Subsequently, by using a mortar and pestle, the plant material was finely grinded in liquid nitrogen and 1 g of powder was resuspended into 4 ml of pre-cold extraction buffer (25 mM Tris-HCl, pH 7.6; 15 mM $MgCl_2$; 150 mM NaCl; 15 mM pNO_2-PhenylPO_4; 60 mM B-glycerophosphate; 0.1 % NO-40 (v/v); 0.1 mM Na3VO_4; 1 mM NaF; 1 mM PMSF; 1 µM E64; 5 % Ethyleenglycol; 100 µM MG132; EDTA-free Ultra Complete proteases inhibitor tablets (Roche)) and mixed well by vortexing. Samples were kept on ice for 20 min and then subjected to sonication for 15 min in dH_20 with ice. Subsequently, cell debris was removed by centrifugation at 4 °C the samples for 15 min. at 14000 rpm. This step was repeated 4 times, while the supernatant was always recovered without disturbing the pellet. Next, the immunoprecipitation step was carried out by dividing the initial 4 ml supernatant into two 1.5 ml Eppendorf tubes to be incubated separately with 50 µl of either Anti-Myc (Miltenyi Biotec, ref. 130-091-123) or Anti-HA (Miltenyi Biotec, ref. 130-091-122) microbeads for 1 h at 4 °C under continuous rotation. 600 µl of the initial supernatant was kept apart to be used as input. Protein complexes were isolated using MACS columns (Milteny Biotec, ref. 130-042-701) as suggested by the manufacturer. Finally, proteins were eluted from the columns by adding pre-heated elution buffer (50 mM Tris-HCL, pH 6.8; 50 mM DTT; 1% SDS; 1 mM EDTA; 0.005% bromophenol blue; 10% glycerol) at 95°C. Coimmunoprecipitated proteins were analyzed by western blotting as described above (see "Protein extraction and immunoblot analysis"). Anti-MYC (Invitrogen, ref. 46-0603; 1:1000) and Anti-HA (Sigma, ref.H6908; 1:1000) primary antibodies were used for western blotting detection.

Protein localization by transient expression in *Nicotiana benthamiana*

Full-coding sequence of *RAPTOR1B* including the stop codon was cloned into *pE3n* vector via *In-Fusion®* (Addgene, https://www.addgene.org). In the *pE3n*, 6XMyc was replaced via *In-Fusion®* cloning by *mTurquose* to generate *mTurquoise-RAPTOR1B*. For *GIGANTEA* (*GI*), conding sequence without stop codon was cloned into *pE2C* vector via *In-Fusion®* (Addgene, https://www.addgene.org). HA tag downstream of *GI* was replaced by mNeonGreen using *In-Fusion®* to generate *GI-mNeonGreen*. Both *mTurquose-RAPTOR1B* and *GI-mNeonGreen* were separately recombined via Gateway cloning into the plant destination vector *pMDC32-HPB* to produce *Pro35S::mTurquose-RAPTOR1B* and *Pro35S::GI-mNeonGreen* (Addgene, https://www.addgene.org). Both constructs were transformed into *A. tumefaciens* for further infiltration in *N. benthamiana*. For signal detection, mTurquose was excited by a 405 nm Diode laser and signal was recovered between 455-508 nm. mNeonGreen was excited by a 488 nm Argon laser and signal was recovered between 505-560 nm. Images were acquired with the same settings for the empty vector control and respective expression constructs. Primers for cloning are listed in Table S1.

Data visualization and statistical test

Details about the statistical tests and parameters are indicated in the legend of each figure. Using RStudio version 1.4.1717, one-way ANOVA and Student's *t-test* were performed employing the R Stats package (https://stat.ethz.ch/R-manual/R-devel/library/stats/html/00Index.html). The "Agricolae"

package was used for the post-hoc Tukey’s test (https://cran.r-project.org/web/packages/agricolae/index.html). All plots were generated with the help of the “ggplot2” package (https://cran.r-project.org/web/packages/DescTools/index.html).

Supplementary Figures

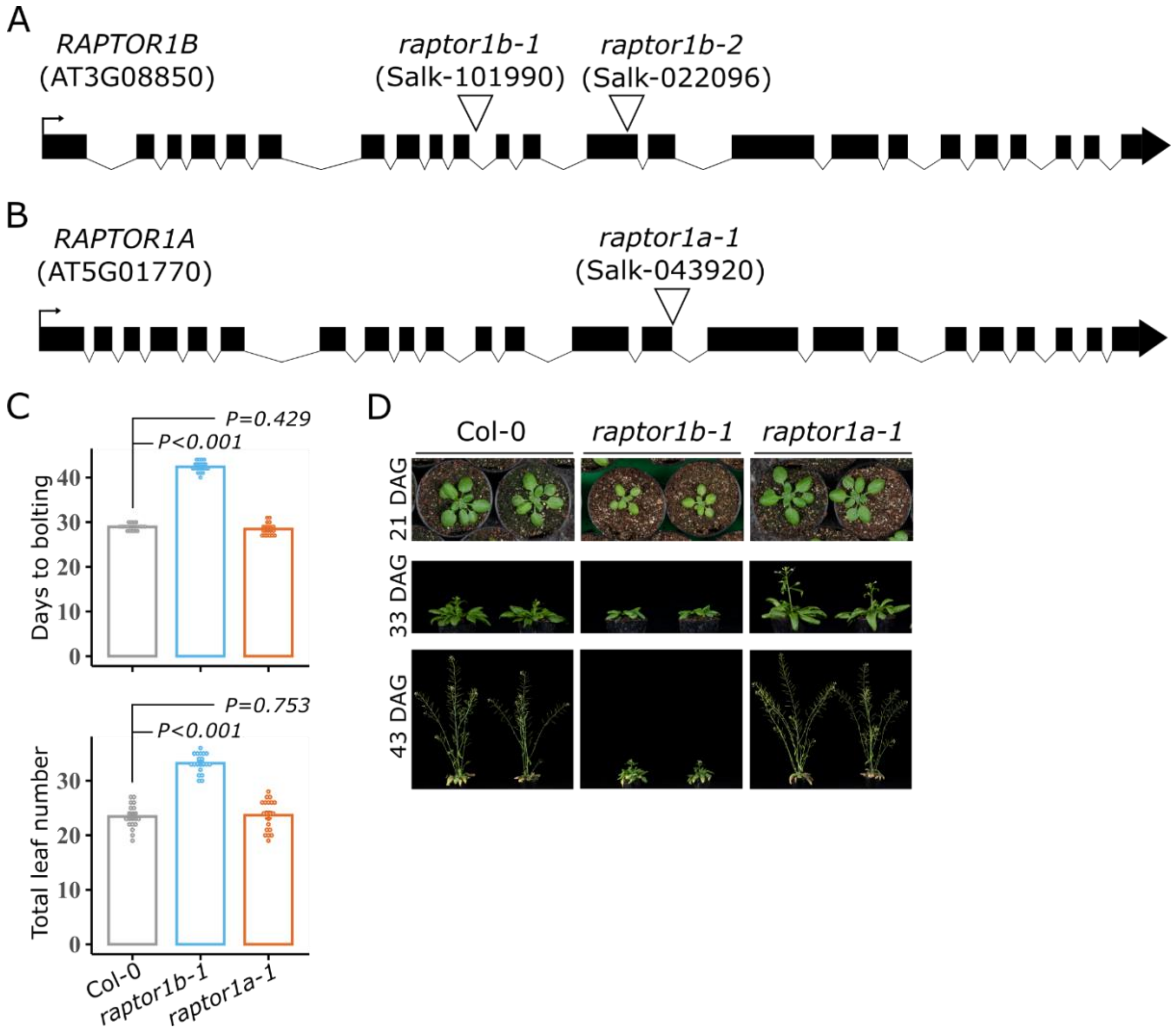

Fig. S1. Flowering time recorded for plants with a mutant allele of *RAPTOR1A* and sketches of the T-DNA insertion lines used in this study. (A) T-DNA insertion sites for *raptor1b-1* (between exon 10 and 11) and *raptor1b-2* (exon 13) in the *RAPTOR1B* gene. **(B)** T-DNA insertion site for *raptor1a-1* (exon 14) in the *RAPTOR1A* gene. **(C)** Days to bolting and total leaf numbers for Col-0, *raptor1b-1* and *raptor1a-1* grown under LD conditions. Significant differences between the genotypes were determined by two-tailed Student's *t*-test. Each circle represents the data of one individual observation (n= 20). **(D)** Representative images of plants in (C) were taken 21, 33 and 43 days after germination (DAG).

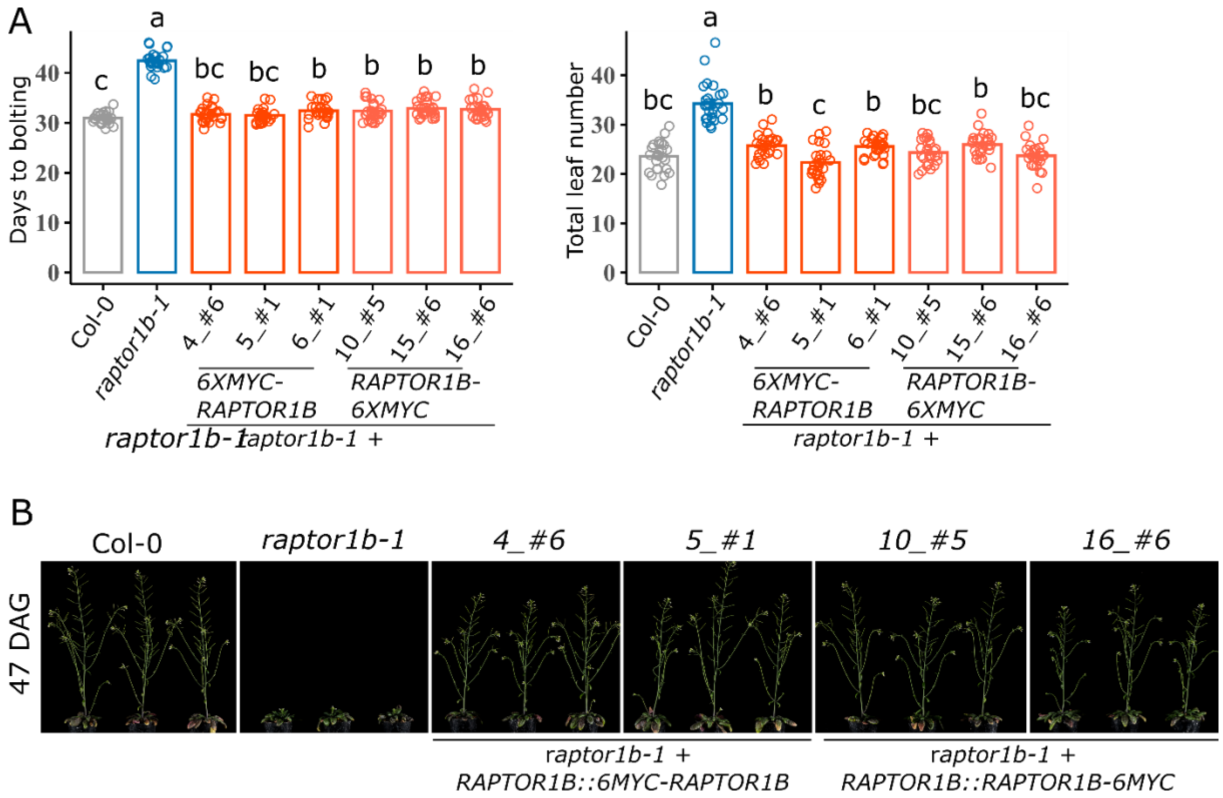

Fig. S2. Complementation of the *raptor1b-1* mutant by stable transformation of *RAPTOR1B::6XMyc-RAPTOR1B* and *RAPTOR1B::RAPTOR1B-6XMyc*. (A) Days to bolting and total leaf numbers for Col-0, *raptor1b-1* and each three independent lines complemented with *RAPTOR1B::6XMyc-RAPTOR1B* (4_#6, 5_#1 and 6_#1) and *RAPTOR1B::RAPTOR1B-6XMyc* (10_#5, 15_#6 and 16_#6) grown under LD conditions. Significant differences among genotypes were determined by one-way ANOVA ($P < 0.05$) followed by a post-hoc Tukey's test as indicated by letters (n=20). Error bars denote SE. **(B)** Representative images of Col-0, *raptor1b-1* and each two independent complemented lines used to determine flowering time in (A). Pictures were taken 47 days after germination (DAG).

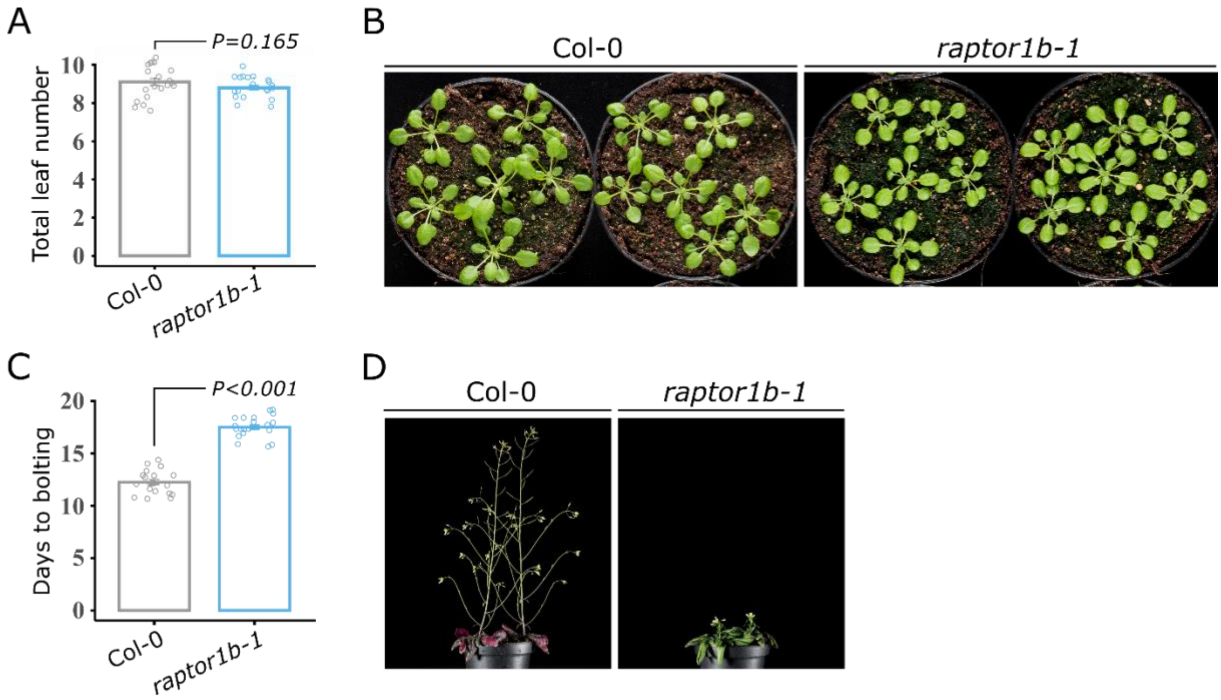

Fig. S3. Phenotypic characterization of Col-0 and *raptor1b-1* plants before and after the photoperiod shift from SD to LD. (A) Total leaf number for wild type and *raptor1b-1* plants after growing under SD conditions for 30 days. Significant differences between the genotypes were determined by two-tailed Student's *t*-test. Each circle represents the data of one individual observation (n= 20). *P* value is shown. Error bars denote SE. **(B)** Representative images of plants in (A) were taken after 30 days in SD and used for counting the number of leaves. **(C)** Days to bolting for Col-0 and *raptor1b-1* upon a photoperiod change from SD to LD conditions. Plants were initially grown for 30 days in SD as in (A). Significant differences between the genotypes were determined by two-tailed Student's *t*-test. Each circle represents the data of one individual observation (n= 20). *P* value is depicted. Error bars denote SE **(D)** Representative images of plants in (C) taken 18 days after the shift from SD to LD.

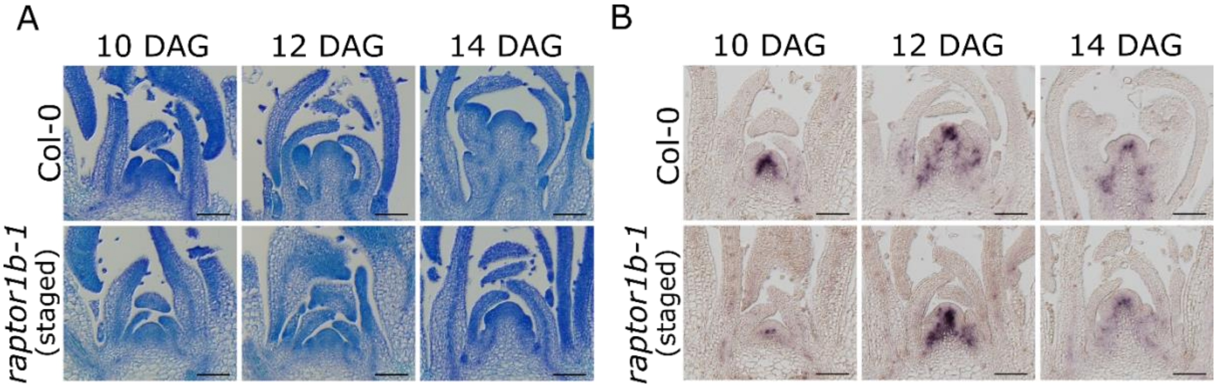

Fig. S4. Longitudinal sections of apices of Col-0 and *raptor1b-1* grown under LD. (A) Longitudinal sections through Col-0 and *raptor1b-1* apices stained with Toluidine blue, for which plants were grown under long day conditions in soil and apices were harvested at 10, 12, and 14 days after germination (DAG). Given that *raptor1b* mutants display late germination (2), *raptor1b-1* seeds were brought to the growth chamber two days in advance (staged) compared to Col-0 seeds, minimizing the effect of late germination at the time of floral transition. Apices were harvested 1 h before dusk. DAG: days after germination. Scale bar: 100 µm. **(B)** RNA *in situ* hybridization using a specific probe for *SOC1* at 10, 12, and 14 DAG. Plants were grown and harvested as described in (A). Bar: 100 µm.

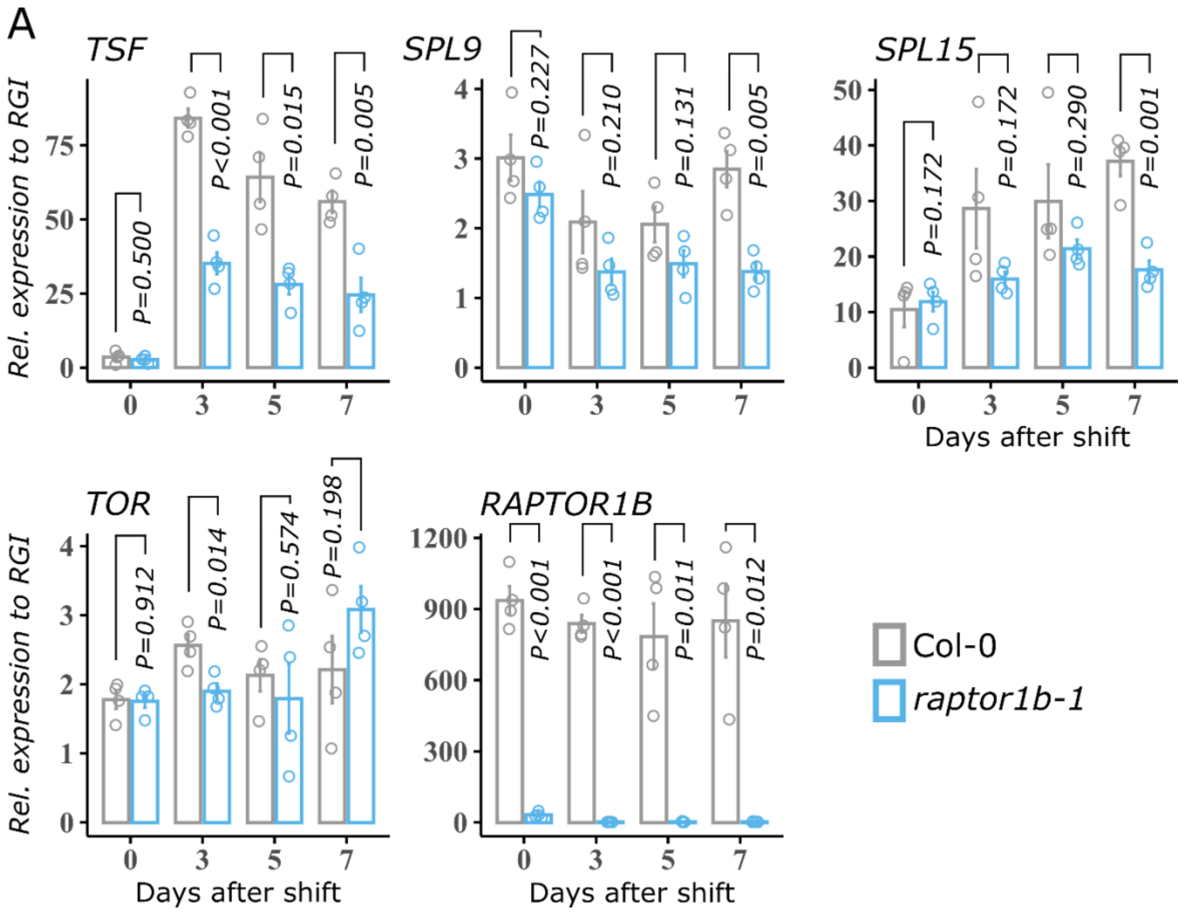

Fig. S5. RAPTOR1B promotes the expression of flowering genes. Supplementary information of Figure 2. (A) Gene expression analysis of *TSF*, *SPL9, SPL15, TOR and RAPTOR1B* in Col-0 and *raptor1b-1* plants by RT-qPCR (see Material and Methods for detailed information about calculation of the relative expression). Growth conditions, plant material, harvesting time points and data analysis are described in Fig. 2A. Significant differences between the genotypes for each day were determined by two-tailed Student's *t*-test (n= 4). *P* values are provided. Error bars denote SE.

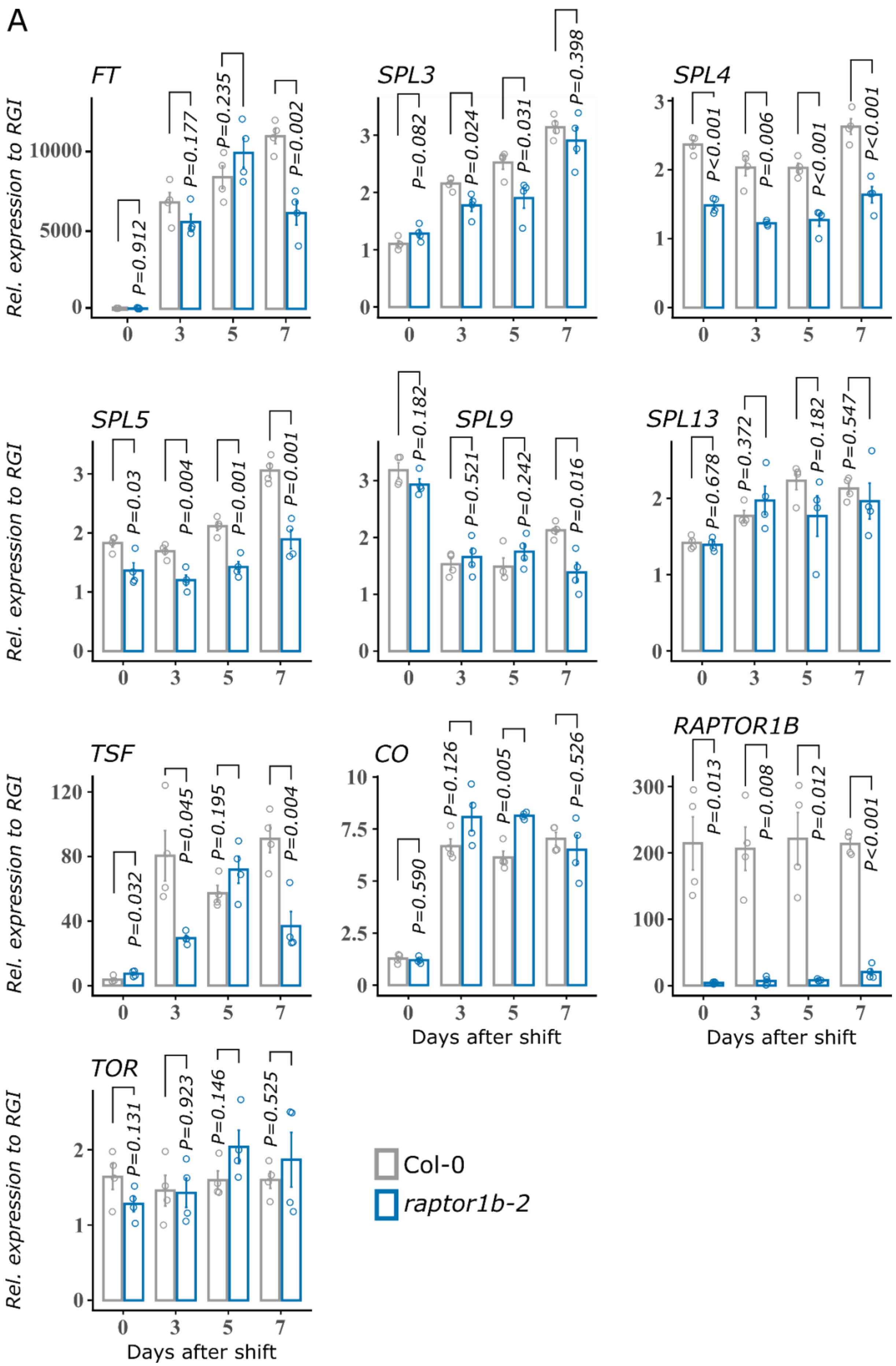

A
FT
SPL3
SPL4
SPL5
SPL9
SPL13
TSF
CO
RAPTOR1B
TOR
Rel. expression to RGI
Days after shift
P=0.912
P=0.177
P=0.235
P=0.002
P=0.082
P=0.024
P=0.031
P=0.398
P<0.001
P=0.006
P<0.001
P<0.001
P=0.03
P=0.004
P=0.001
P=0.001
P=0.182
P=0.521
P=0.242
P=0.016
P=0.678
P=0.372
P=0.182
P=0.547
P=0.032
P=0.045
P=0.195
P=0.004
P=0.590
P=0.126
P=0.005
P=0.526
P=0.013
P=0.008
P=0.012
P<0.001
P=0.131
P=0.923
P=0.146
P=0.525
Col-0
raptor1b-2

407

Fig. S6. Gene expression analysis of selected flowering genes for the second mutant allele of *RAPTOR1B*, *raptor1b-2*, during a photoperiod shift from SD to LD. (A) Expression analysis of *FT, TSF, SPL3, SPL4, SPL5, SPL9, SPL13, CO, TOR* and *RAPTOR1B* in Col-0 and *raptor1b-2* plants by RT-qPCR. Growth conditions, plant material, harvesting time points and data analysis were performed as described in Fig. 2A. Significant differences between Col-0 and *raptor1b-2* for each day were determined by two-tailed Student's *t*-test (n= 4). *P* values are provided. Error bars denote SE.

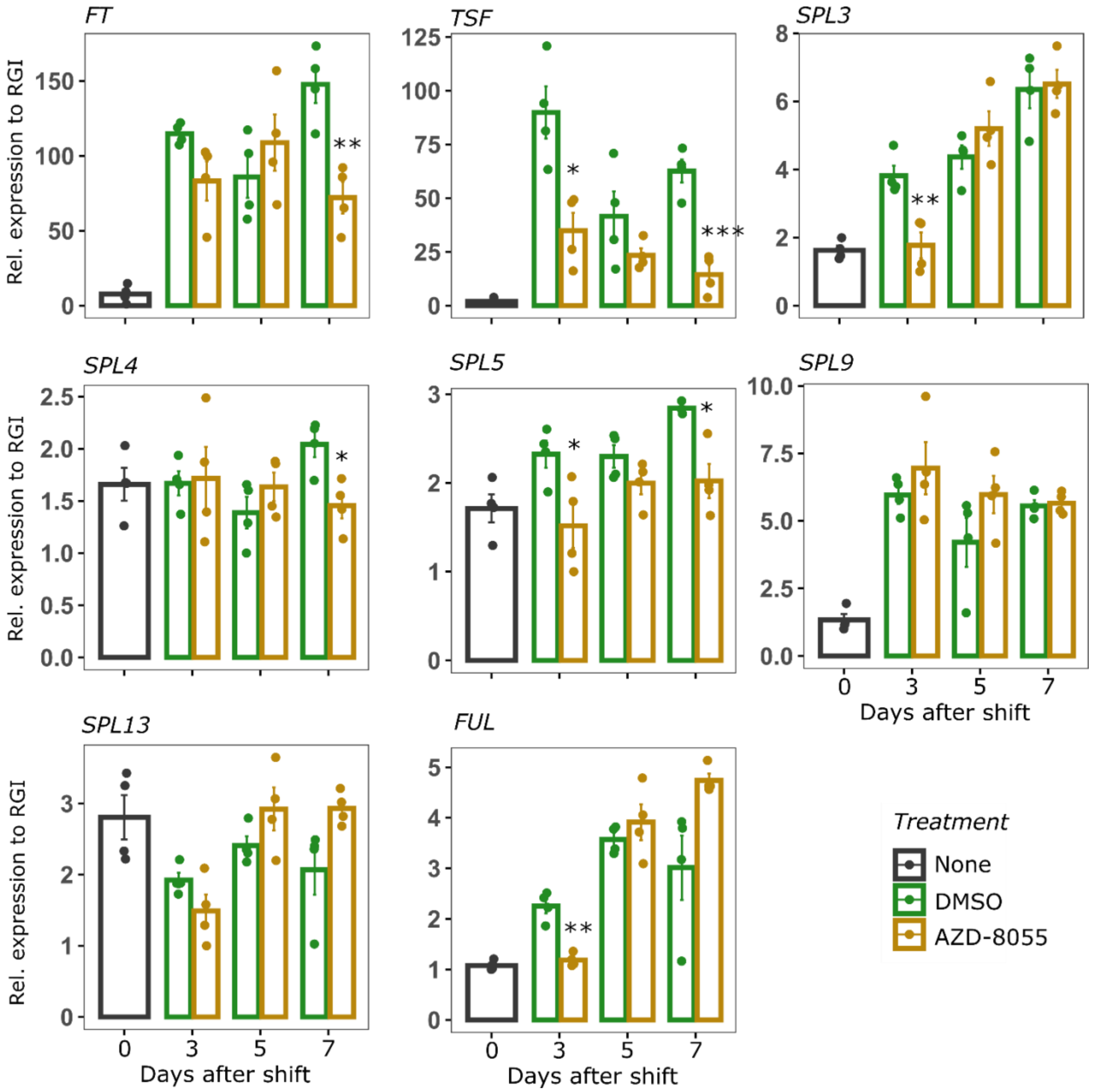

Fig S7. AZD-8055 reduces expression of flowering genes. Gene expression analysis of *FT, TSF, SPL3*, *SPL4*, *SPL5*,*SPL9*, *SPL13* and *FUL* by RT-qPCR. Col-0 plants were grown initially in SD for 30 days and then transferred to LD. AZD-8055 (10 µM) and DMSO (1 % v/v) were sprayed 30 minutes before light was on at days 0, 2, 4, and 6 after the photoperiod shift to LD. Rosettes were harvested at dusk at days 0, 3, 5, and 7. None treatment: plants at 0 DAS not treated with either DMSO or AZD-8055. Significant differences between DMSO and AZD were determined by two-tailed Student's *t*-test (n= 4). *p<0.05, **p<0.01, ***p<0.001. Error bars denote SE.

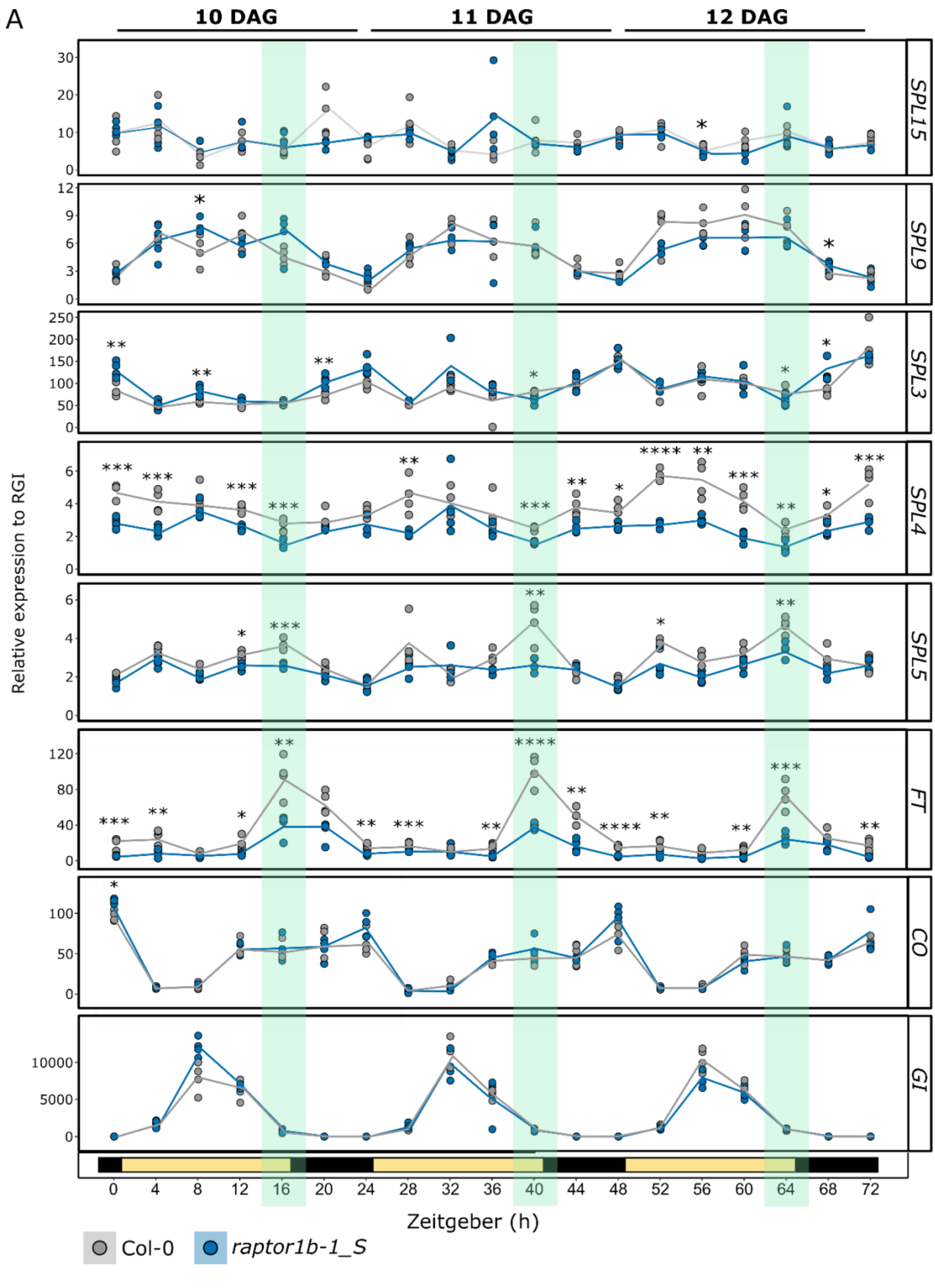

Fig. S8. Diel expression analysis of *FT*, *CO*, *GI,* SPL3, SPL4, SPL5, SPL9 and SPL15 in Col-0 and *raptor1b-1* under LD conditions. (A) Col-0 and *raptor1b-1* plants were grown from the beginning under LD photoperiod. *raptor1b-1* seeds were brought into the growth chamber two days in advance after stratification to compensate for the late germination (1), and therefore, it is referred in the plot as *raptor1b-1*_S (S, staged). Whole rosettes were harvested every 4 h for three consecutive days at 10, 11 and 12 DAG, which is the time when the floral transition occurs (see *SI Appendix*, Fig. S3B and C). Four

biological replicates per time point and genotype were done by harvesting a pool of ~5 rosettes in each case. Relative gene expression was calculated as explained in Fig. 2A. Significant differences between the genotypes for each time point were determined by two-tailed Student's *t-test* (n= 4, *$P<0.05$, **$P<0.01$, ***$P<0.001$, ****$P<0.0001$). The shaded green-field area along the plots highlights the *Zeitgeber* time 16 h (dusk) for each day.

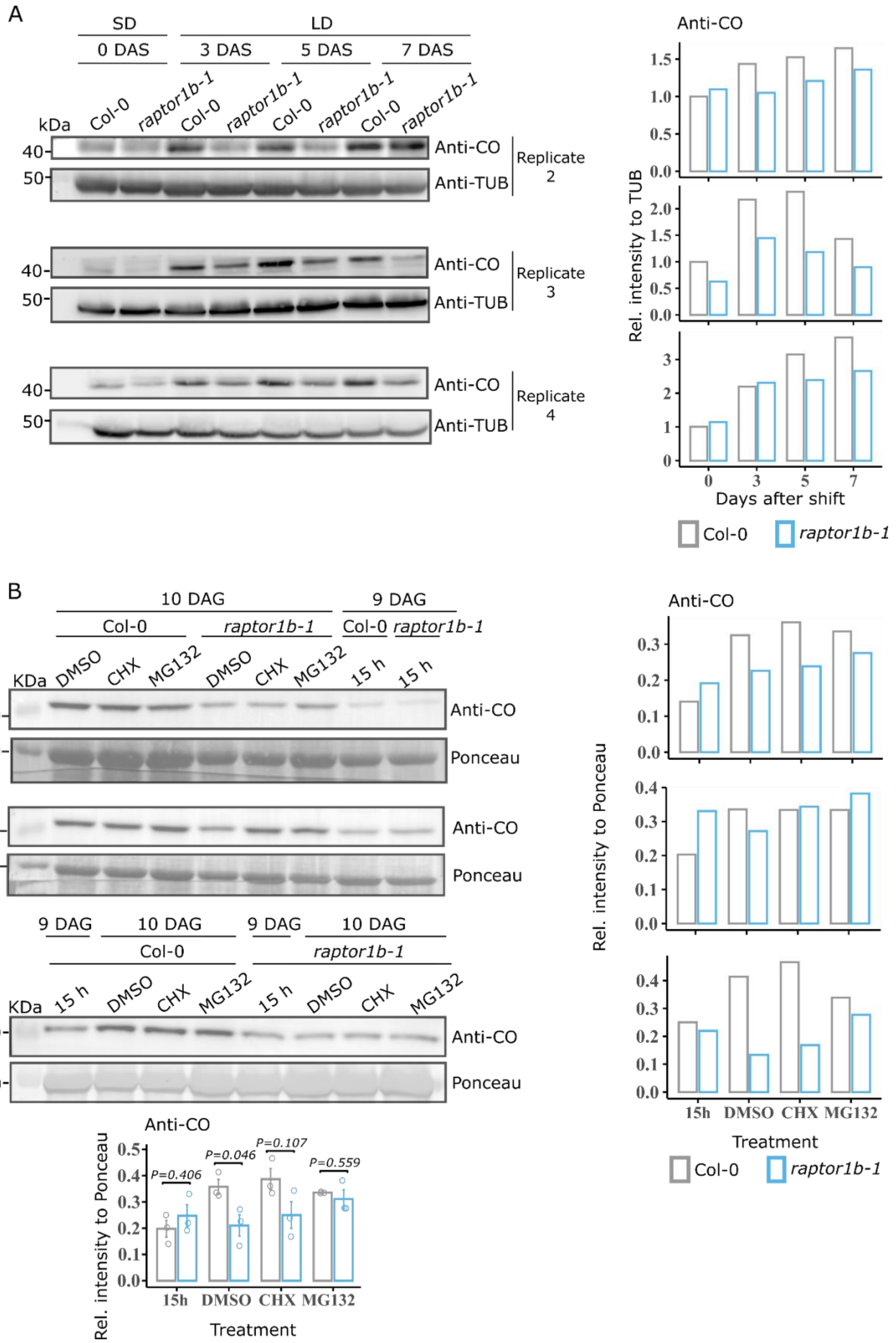

Fig. S9. RAPTOR1B contributes to CONSTANS protein stability under LD during the floral transition. (A) Supplementary information of Fig. 3. Additional replicates of the western blot analysis to determine CONSTANS protein levels described in main Fig. 3A. On the right panel, relative CONSTANS

protein levels correspond to each of the replicates on the left side, and the relative intensities were calculated as described in Fig. 3B. **(B)** Blocking protein degradation in *raptor1b-1* mutant increases CO levels comparable to Col-0. Western blot analysis of CO levels in Col-0 and *raptor1b-1* upon treatment with Cycloheximide (CHX, 100µM) or MG132 (100 µM). Relative CONSTANT levels for each replicate are depicted on the right panel. Below the blots, a plot combining the single replicates with statistical analysis is depicted. Plants were germinated and grown under LD in a hydroponic system for 10 days. Inhibitors and DMSO (1% v/v, mock treatment) were added 30 minutes before dawn. Entire rosettes were collected 15 h after the treatment on day 10. Shoots harvested on day 9 before the start of the treatment were used as control. Endogenous CO was immuno-detected with Anti-CO. A Ponceau stain was used as the loading control. Anti-CO signal was normalized to the respective Ponceau loading control to determine the relative intensity for each treatment. Significant differences between the genotypes for each treatment were determined by a two-tailed Student's *t*-test (n= 3). Respective *P* values are provided. Error bars denote SE

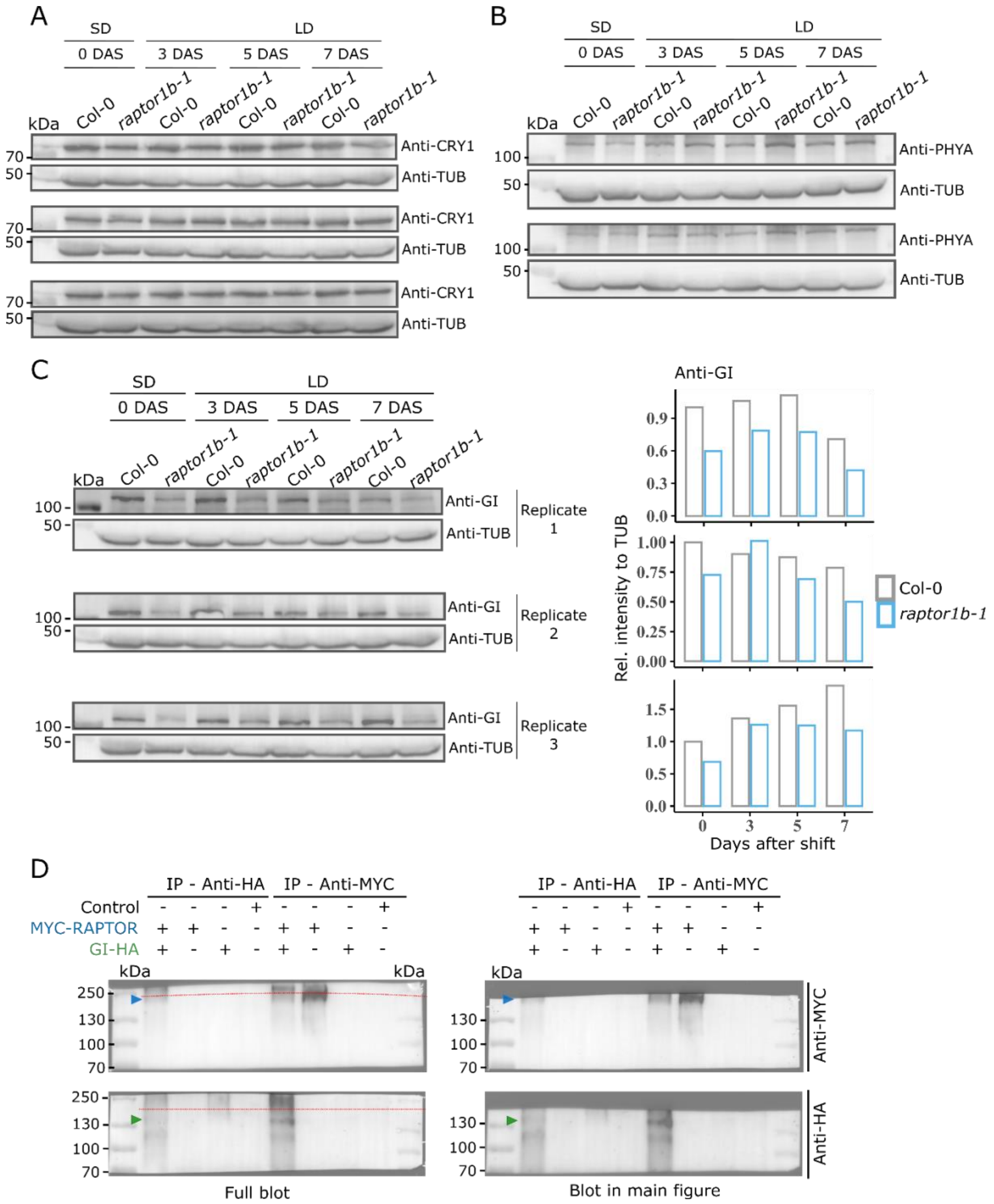

Fig. S10. Western blot analysis to determine protein levels of CRYPTOCHROME 1 (CRY1), PHYTOCHROME A (PHYA), and GIGANTEA (GI) in Col-0 and *raptor1b-1* during a SD to LD photoperiod shift. (A) Western blot for CRY1. Grow conditions and plant material are the same as described in Fig. 2A. Proteins were extracted and endogenous CRY1 and TUBULIN proteins were immuno-detected using Anti-CRY1 and Anti-TUB, respectively. Three biological replicates are depicted. **(B)** Western blot for PHYA. Grow conditions and plant material are the same as described in Fig. 2A. Proteins were extracted and endogenous PHYA and TUBULIN proteins were immuno-detected using Anti-PHYA and Anti-TUB, respectively. Two biological replicates are depicted. **(C)** Additional replicates of the western blot analysis to determine GIGANTEA (GI) protein levels described in main Fig. 4A. On the right panel, relative GI levels correspond to each of the replicates on the left side and the relative intensities were calculated as described in Fig. 4B. **(D)** Full blots for main Figure 4E. Dotted lines show where the blots were cut to improve the signal detection of week bands.

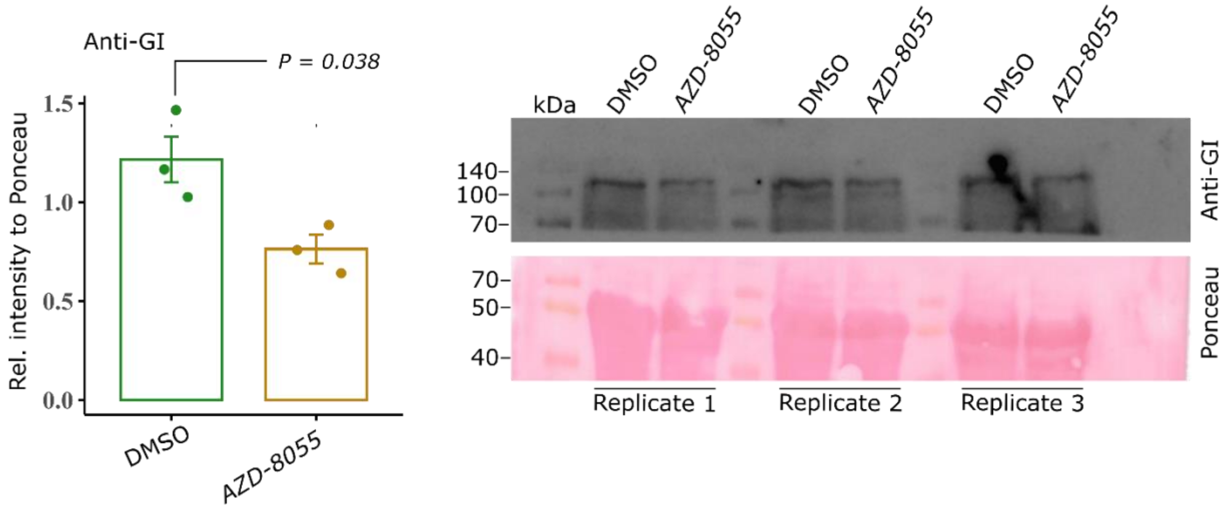

Fig. S11. AZD-8055 reduces GI protein levels during the flower transition. Western blot analysis of GI levels in Col-0 upon treatment with AZD-8055. Plants were germinated and grown under LD in a hydroponic system for 10 days. DMSO (1 % v/v) and AZD-8055 (2 µM) were added 30 minutes before dawn. Entire rosettes were collected 15 h after the treatment at day 10. Endogenous GI was immuno-detected with Anti-GI. Relative CO levels were calculated as follows: Anti-GI signal was normalized to the respective Ponceau loading control to determine the relative intensity for each treatment. Significant differences between DMSO and AZD were determined by two-tailed Student's *t*-test (n= 3). Error bars denote SE.

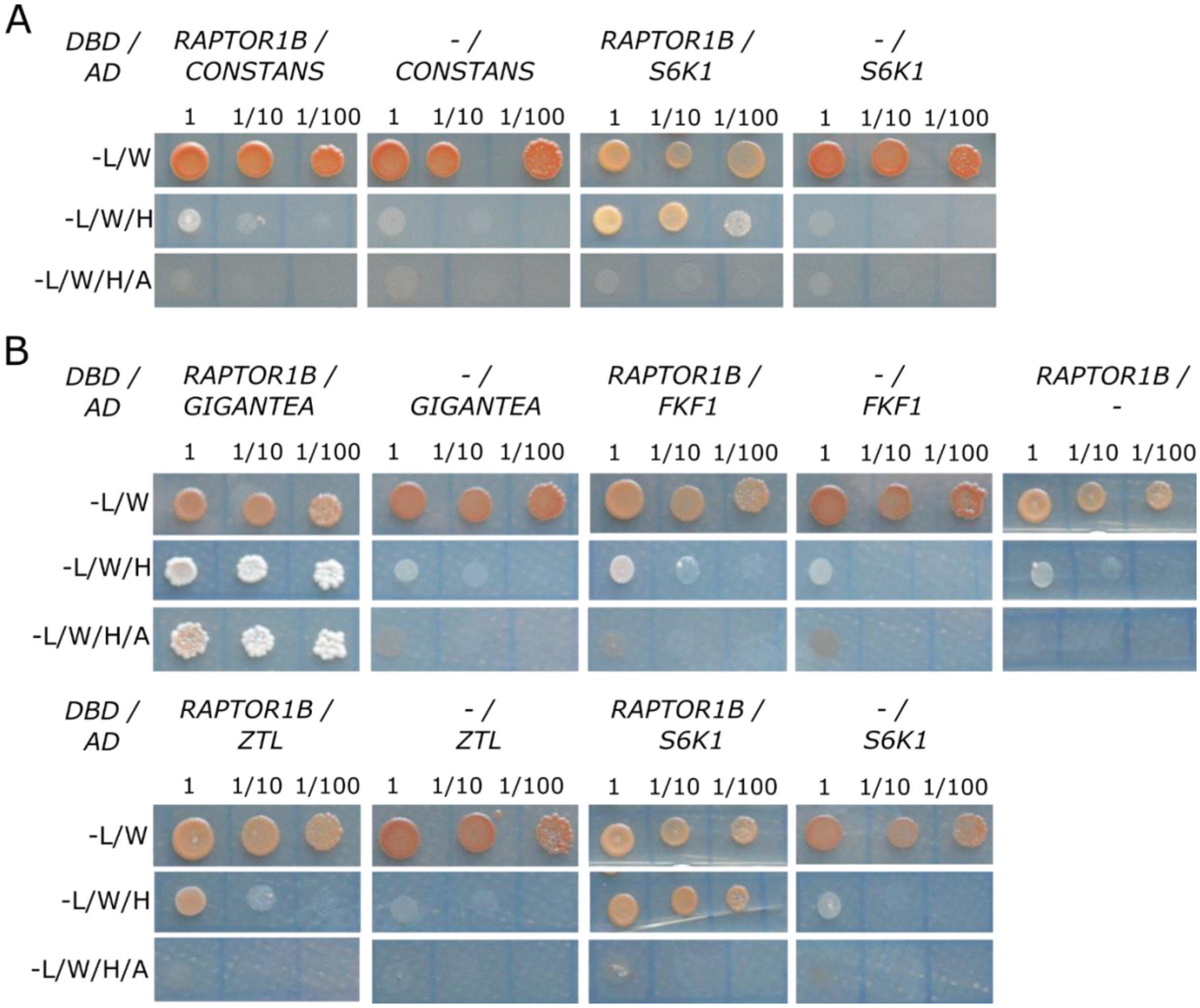

Fig. S12. Yeast 2 hybrid assays to test additional interactions of RAPTOR1B, including the positive control with 40S Ribosomal protein S6 Kinase 1 (S6K1). (A) Full coding sequence of *RAPTOR1B* was cloned into *pDEST*^{TM}*32* (harboring the DNA binding domain, DBD). Full-coding sequence of *CO* and a cDNA fragment from amino acid 9 to 293 in case of *S6K1*, were cloned into *pDEST*^{TM}*22* (containing the activation domain, AD). *pDEST*^{TM}*32* and *pDEST*^{TM}*22* constructs were cotransformed into yeast as described elsewhere (14). **(B)** *RAPTOR1B* and *S6K1* were cloned as in (A). Full coding sequences of *GIGANTEA*, *FKF1*, and *ZTL* were cloned into *pDEST*^{TM}*22*. Constructs were cotransformed as in (A). In (A) and (B), as controls, respective empty *pDEST*^{TM}*32* lacking *RAPTOR1B* or *pDEST*^{TM}*22* lacking the other proteins were simultaneously cotransformed and spotted in the same plate. The different combinations of plasmids were spotted on synthetic double (- L/W), triple (- L/W/H) or quadruple (- L/W/H/A) dropout medium. L (Leucine), W (Tryptophan), H (Histidine), A (Adenine).

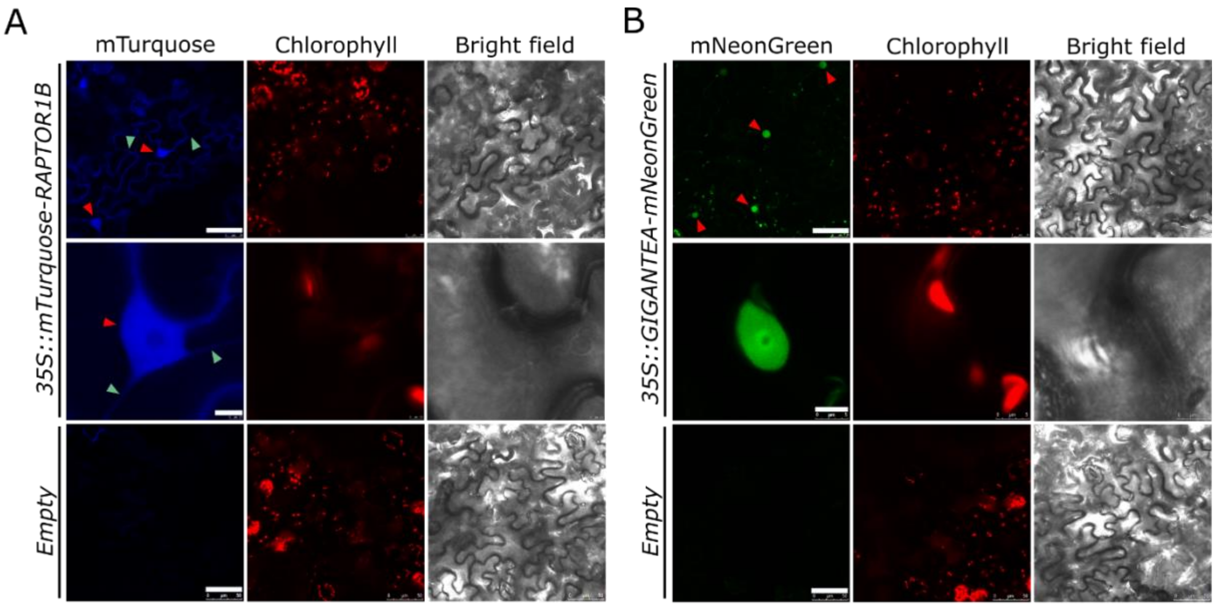

Fig. S13. Subcellular localization of RAPTOR1B and GIGANTEA. (A) Transient expression of *Pro35S::mTurquoise-RAPTOR1B* in *N. benthamiana* leaves. Upper panel depicts mTurquoise signal in nuclei (red triangles) and in cytoplasm (green triangles). Middle panel displays a closer view of a selected nucleus (red triangle) and cytoplasmic string are also visible (green triangle). Bottom panel corresponds to infiltration with the empty vector backbone (*pMDC32-HPB*). All images were acquired using the same settings for detecting mTurquoise signal. **(B)** Transient expression of *Pro35S::GI-mNeonGreen* in *N. benthamiana* leaves. Upper panel depicts mNeonGreen signal in nuclei (red triangles). Middle panel displays a closer view of a selected nucleus. Bottom panel corresponds to infiltration with the empty vector backbone (*pMDC32-HPB*). All images were acquired using the same settings for detecting mNeonGreen signal. Scale bars for (A) and (B), upper panels: 50 µm, middle panels: 5 µm, bottom panels: 50 µm.

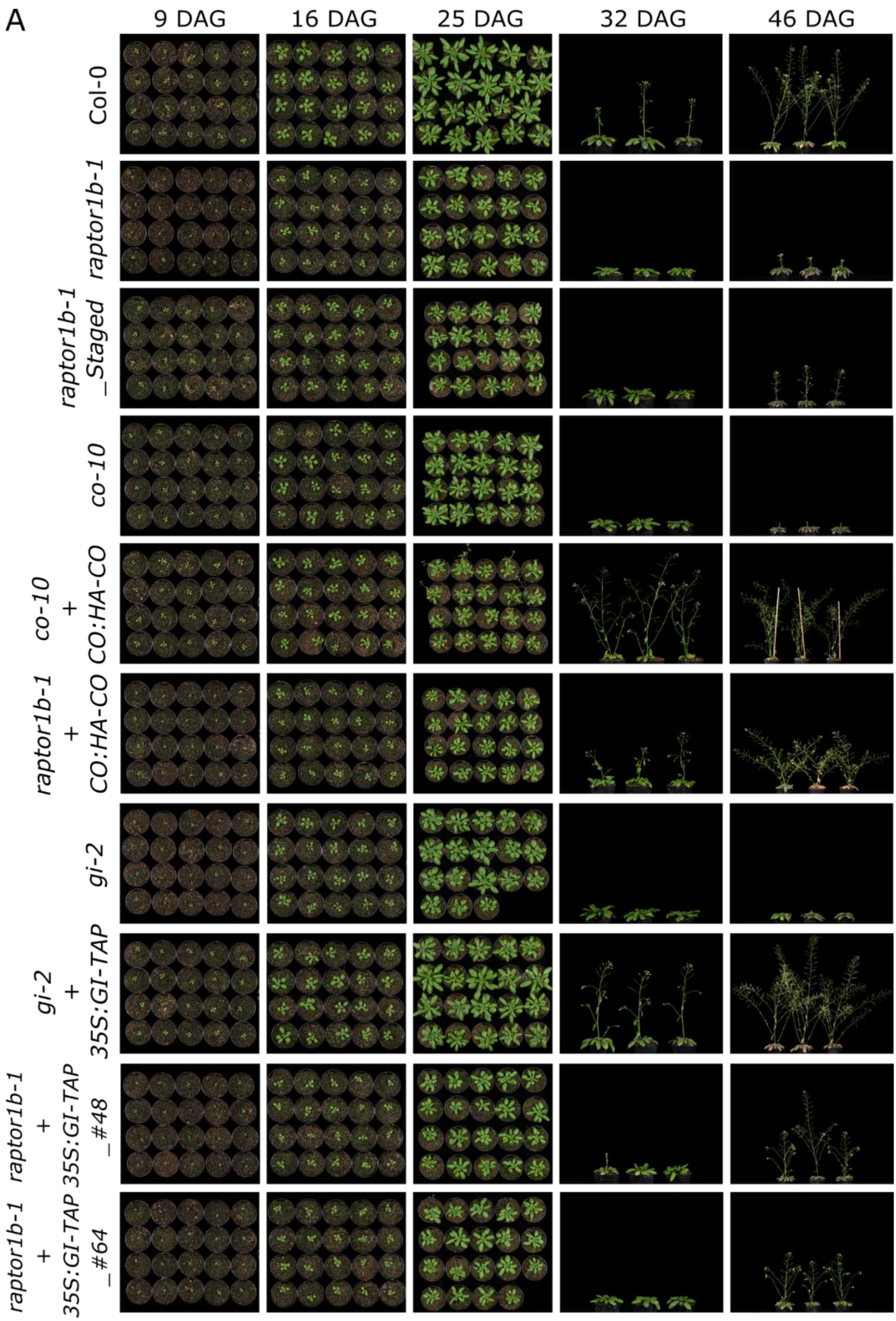

Fig. S14. (A) Images corresponding to the flowering time experiment in Fig. 5A. Images were taking 9, 16, 25, 32, and 46 after germination (DAG).

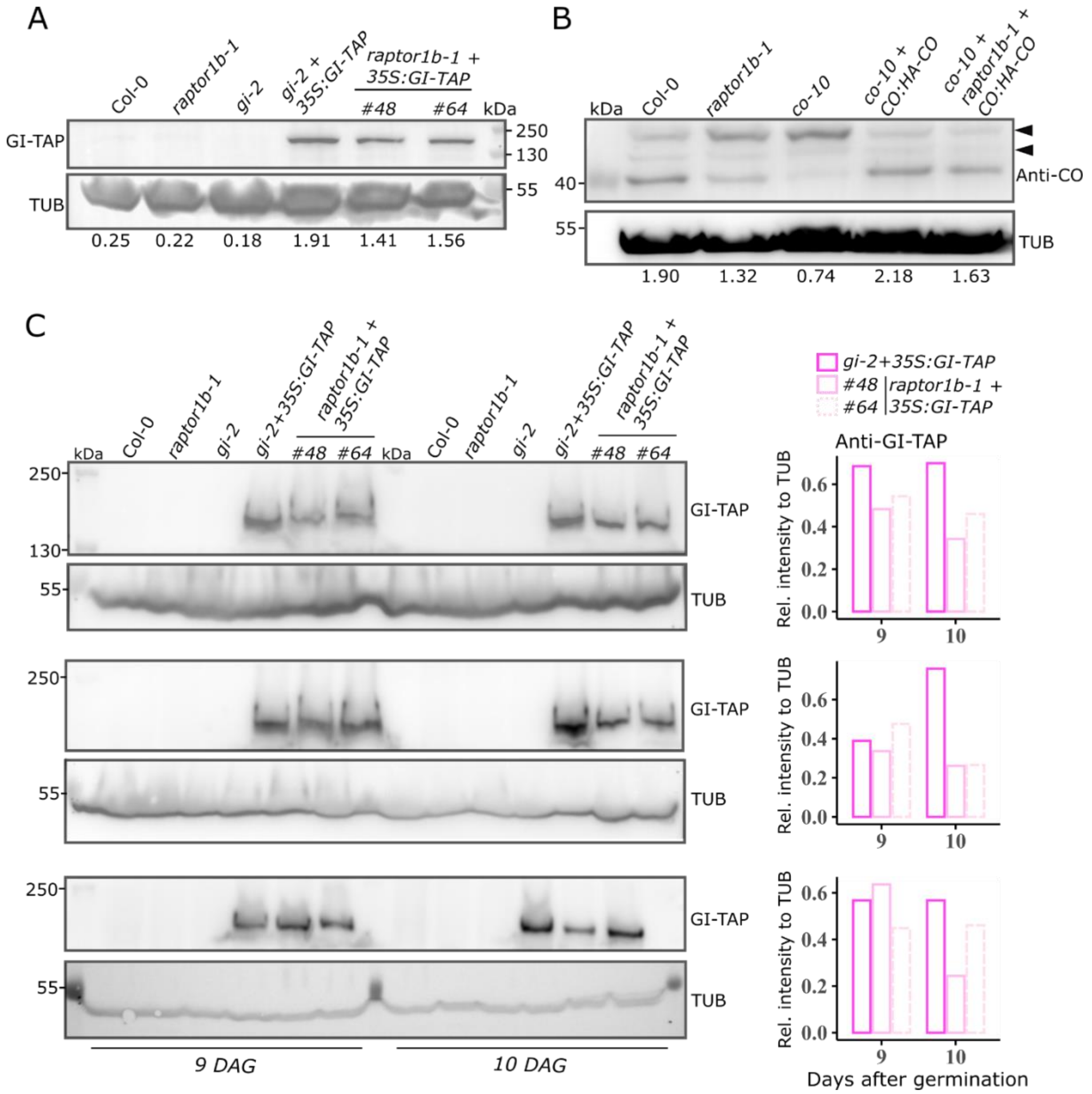

Fig. S15. Western blot analyses for GIGANTEA and CONSTANS at bolting and during the floral transition. (A) Western blot analysis for GI-TAP (~150 kDa). Plant material corresponds to flowering time experiment of main Fig. 5A. At bolting, for each genotype, a pool of three rosettes were collected 15 h after the onset of light. Proteins were extracted and GI-TAP and TUBULIN were immuno-detected using Anti-GI and Anti-TUB, respectively. Anti-GI signal was normalized to the respective Anti-TUB signal to determine the relative intensity for each genotype and the respective numbers are depicted below the blots. **(B)** Western blot analysis for CONSTANS (CO) (endogenous CO ~42 kDa; HA-CO ~43 kDa). Plant material corresponds to main Fig. 5A and protein extraction was performed as described in (A). CO/HA-CO and TUBULIN were immuno-detected using Anti-CO and Anti-TUB, respectively. Anti-CO signal was normalized to the respective Anti-TUB signal to determine the relative intensity for each genotype and the respective numbers are depicted below the blots. Dark triangles depict unspecific bands. Note: HA-tag adds 1.1 kDa to endogenous CO. **(C)** Additional replicates of the western blot analysis described in main Fig. 5B and C. On the right panel, relative GI levels correspond to each of the replicates on the left side and the relative intensities were calculated as described in Fig. 5C.

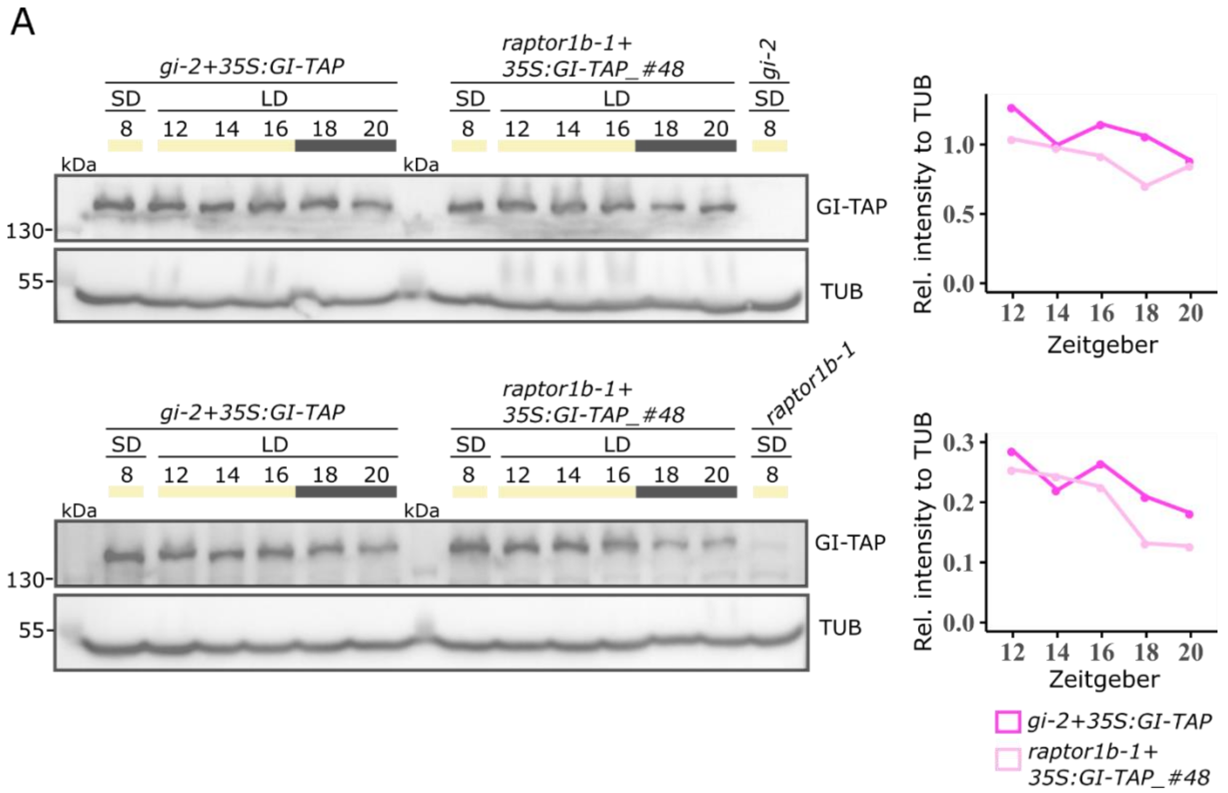

Fig. S16. RAPTOR1B functions upstream of GI to contribute to CO stability at dusk and thus promote the floral transition in Arabidopsis. Supplementary information of Fig. 5D. (A) Additional replicates of main Fig. 5D and $. For these replicates, instead of Col-0, proteins were extracted from *gi-2* or *raptor1b-1* mutants to be used as controls, respectively. The two plots on the right correspond to the relative GI-TAP levels calculated from the blots on the left panel as described in main Fig. 5E.

Table S1. Primer list

AGI /Gene bank	Primer Name	Sequence	Purpose
	BP	attttgccgatttcggaac	Genotyping SALK lines
	LB-1	tagcatctgaatttcataaccaatctcgatacac	Genotyping SAIL lines
AT3G08850	RAPTOR B LP_3	agcagtgacggatacaattgc	Genotyping *raptor1b-1* (SALK_101990)
AT3G08850	RAPTOR B RP_3	gttgtttgcagaagcttcagc	Genotyping *raptor1b-1* (SALK_101990)
AT3G08850	RAPTOR B LP_4	caatatgaagctgcggctaac	Genotyping *raptor1b-2* (SALK_022096)
AT3G08850	RAPTOR B RP_4	catcggatcaagttgcttacc	Genotyping *raptor1b-2* (SALK_022096)
AT5G01770	ra1_LP	aagaggatgagcgaactaggc	Genotyping *raptor1a-1* (SALK_043920)
AT5G01770	ra1_RP	tttgtcttggaaagttgtggg	Genotyping *raptor1a-1* (SALK_043920)
At5g15840	co_LP	aagctgttgtgacacatgctg	Genotyping *co-10* (SAIL_24_H04)
At5g15840	co_RP	ccccttctttcagataccagc	Genotyping *co-10* (SAIL_24_H04)
AT1G22770	gi-2_Fw_seq500bp	ttcatgtcttattga	Genotyping/sequencing for gi-2 deletion
AT1G22770	gi-2_Rv_seq500bp	aaataaaagaaggag	Genotyping/sequencing for gi-2 deletion
AT3G08850	RAPTORB_Forw_pE3n	cggaagcccgggatccatggcattaggagacttaatggtg	Cloning ProRAPTOR1B::6XMyc-RAPTOR1B, Coimmunoprecipitation and subcellular localization
AT3G08850	RAPTORB_Rev_pE3n	gctacttatgcggccgctcatcttgcttgcgagttgtc	Cloning ProRAPTOR1B::6XMyc-RAPTOR1B,coimmunoprecipitation and subcellular localization
AT3G08850	RAPTORB_Forw_pE3c	tcagtcgactggatccatggcattaggagacttaatggtg	Cloning ProRAPTOR1B::RAPTOR1B-6XMyc
AT3G08850	RAPTORB_Rev_pE3c	atagcttgtgcggccgctcttgcttgcgagttgtc	Cloning ProRAPTOR1B::RAPTOR1B-6XMyc
AT3G08850	promRAP fwB	accaattcaggtcgacgatgcgagattcccaagtgagagag	Cloning ProRAPTOR1B (Promoter)
AT3G08850	promRAP revB	aatcgataccgtcgacgaaatcacgcaaatcaaatcctcagcc	Cloning ProRAPTOR1B (Promoter)
AT3G08850	RAPTORB_cDNA_Forw	ggggacaagtttgtacaaaaaagcaggcttttggatggcattaggagacttaatg	Cloning for Yeast two hybrid
AT3G08850	RAPTORB_cDNA_Rev	ggggaccactttgtacaagaaagctgggtcgactcatcttgcttgcgagttgt	Cloning for Yeast two hybrid
At5g15840	CO_cDNA_Forw	ggggacaagtttgtacaaaaaagcaggctttatgttgaaacaagagagtaacgaca	Cloning for Yeast two hybrid
At5g15840	CO_cDNA_Rev	ggggaccactttgtacaagaaagctgggtatcagaatgaaggaacaatcccata	Cloning for Yeast two hybrid
AT1G22770	GI_cDNA_Forw	ggggacaagtttgtacaaaaaagcaggctggatggctagttcatcttcatctga	Cloning for Yeast two hybrid
AT1G22770	GI_cDNA_Rev	ggggaccactttgtacaagaaagctgggtgttattgggacaaggatatagtacagcc	Cloning for Yeast two hybrid
AT1G68050	FKF1_cDNA_Forw	ggggacaagtttgtacaaaaaagcaggctttatggcgagagaacatgcga	Cloning for Yeast two hybrid

AT1G68050	FKF1_cDNA_Rev	ggggaccactttgtacaagaaagctgggttttacagatccgagtcttgccg	Cloning for Yeast two hybrid
AT5G57360	ZTL_cDNA_Forw	ggggacaagtttgtacaaaaaagcaggctttatggagtgggacagtggttc	Cloning for Yeast two hybrid
AT5G57360	ZTL_cDNA_Rev	ggggaccactttgtacaagaaagctgggttctaatgaggaagaaagaagaagaagga	Cloning for Yeast two hybrid
AT3G08730	S6K1_Forw_Peptide	ggggacaagtttgtacaaaaaagcaggctttcccaacaaaatccagaaacagc	Cloning for Yeast two hybrid
AT3G08730	S6K1_Rev_Peptide	ggggaccactttgtacaagaaagctgggtctggctatccgcacatggagtttgatc	Cloning for Yeast two hybrid
AT1G22770	GI_HA_C_For	tcagtcgactggatccatggctagttcatcttcatctgag	Cloning for Coimmunoprecipitation and subcellular localization
AT1G22770	GI_HA_C_Rev	cctccgcttgcggccgcttgggacaaggatatagtacagccg	Cloning for Coimmunoprecipitation and subcellular localization
At5g20700	gDNA FLZ14 N fw	ggccgctggggccatgcttactaaaagaacccatc	Cloning for Coimmunoprecipitation (Control)
At5g20700	attB1 mNEON C FLZ14 fw	ggggacaagtttgtacaaaaaagcaggcttaatgcttactaaaagaacccatcccatga	Cloning for Coimmunoprecipitation (Control)
UEC50308.1	mNeonGreen tag F	ggggacaagtttgtacaaaaaagcaggcttaatggtgagcaagggagagga	Cloning for Coimmunoprecipitation (Control)
UEC50308.1	mNeonGreen tag R	ttttagtaagcatggccccagcggccgca	Cloning for Coimmunoprecipitation (Control)
QBQ65841.1	Tourq_For_N_2_RAPTOR	accaattcaggtcgacatggtgagcaagggcgagga	Cloning for subcellular localization
QBQ65841.1	Tourq_Rev_N_2_RAPTOR	tgccatggatcccggggaggccccagcggccgca	Cloning for subcellular localization
UEC50308.1	mNeon_For_C_2_GI	ttgtcccaagcggccgcaggctcgggaggtggagg	Cloning for subcellular localization
UEC50308.1	mNeon_Rev_C_2_GI	gaaagctgggtctagatttgtatagctcgtccattcccatc	Cloning for subcellular localization
At1G65480	FT_F	tggaacaacctttggcaatgag	qRT-PCR
At1G65480	FT_R	cgacacgatgaattcctgcag	qRT-PCR
AT4G20370	TSF_F	ctcgggaattcatcgtattg	qRT-PCR
AT4G20370	TSF_R	ccctctggcagttgaagtaa	qRT-PCR
AT2G33810	SPL3_F	gagtttgtcaggtcgagagttgtacc	qRT-PCR
AT2G33810	SPL3_R	gcagactttgtgtcgtttgtggt	qRT-PCR
AT1G53160	SPL4_F	aatggtcaggtggtgatgcag	qRT-PCR
AT1G53160	SPL4_R	gcataggaagtgtcatctctaccctt	qRT-PCR
AT3G15270	SPL5_F	cagcaggtttcatgagctaccag	qRT-PCR
AT3G15270	SPL5_R	caaaactgtcaccagagatcttcctc	qRT-PCR
AT2G42200	SPL9_F	cttcgctttacgaaaatggtgatg	qRT-PCR
AT2G42200	SPL9_R	actggccgcctcatcactct	qRT-PCR
AT3G57920	SPL15_F	catctctttacggaaaccccaatg	qRT-PCR
AT3G57920	SPL15_R	gccgctgcatcactgatctt	qRT-PCR
AT1G22770	GI_F	agcagtggtcgacggtttatc	qRT-PCR
AT1G22770	GI_R	atgggtatggagctttggttc	qRT-PCR
At5g15840	CO_F	aacagcttcacacccaagaacg	qRT-PCR
At5g15840	CO_R	ggtcaggttgttgctctactg	qRT-PCR
AT3G08850	RAPTOR1B_F	tcaatccagggtcacaagcc	qRT-PCR
AT3G08850	RAPTOR1B_R	gatgcactcaccacctttgc	qRT-PCR
AT1G50030	TOR_F	catctgcgcgtctggaaatg	qRT-PCR
AT1G50030	TOR_R	cctcgtcgtactttgccctt	qRT-PCR
AT1G13320	PDF2_F	taacgtggccaaaatgatgc	qRT-PCR
AT1G13320	PDF2_R	gttctccacaaccgcttggt	qRT-PCR
AT4G34270	TIP41_F	gtgaaaactgttggagagaagcaa	qRT-PCR

AT4G34270	TIP41_R	tcaactggataccctttcgca	qRT-PCR
AT4G27960	UBC9_F	tcacaatttccaaggtgctgc	qRT-PCR
AT4G27960	UBC9_R	tcatctgggtttggatccgt	qRT-PCR
AT4G26410	RHIP_F	gagctgaagtggcttccatgac	qRT-PCR
AT4G26410	RHIP_R	ggtccgacatacccatgatcc	qRT-PCR

SI References

1. M. A. Salem, Y. Li, A. Wiszniewski, P. Giavalisco, Regulatory-associated protein of TOR (RAPTOR) alters the hormonal and metabolic composition of Arabidopsis seeds, controlling seed morphology, viability and germination potential. *Plant Journal* **92**, 525–545 (2017).
2. M. A. Salem, *et al.*, RAPTOR Controls Developmental Growth Transitions by Altering the Hormonal and Metabolic Balance. *Plant Physiol* **177**, 565–593 (2018).
3. G. H. Anderson, B. Veit, M. R. Hanson, The Arabidopsis AtRaptor genes are essential for post-embryonic plant growth. *BMC Biol* **3**, 1–11 (2005).
4. D. Deprost, H. N. Truong, C. Robaglia, C. Meyer, An Arabidopsis homolog of RAPTOR/KOG1 is essential for early embryo development. *Biochem Biophys Res Commun* **326**, 844–850 (2005).
5. T. Araki, Y. Komeda, Analysis of the role of the late-flowering locus, GI, in the flowering of Arabidopsis thaliana. *Plant Journal* **3**, 231–239 (1993).
6. S. Fowler, *et al.*, GIGANTEA: A circadian clock-controlled gene that regulates photoperiodic flowering in Arabidopsis and encodes a protein with several possible membrane-spanning domains. *EMBO Journal* **18**, 4679–4688 (1999).
7. K. M. David, U. Armbruster, N. Tama, J. Putterill, Arabidopsis GIGANTEA protein is post-transcriptionally regulated by light and dark. *FEBS Lett* **580**, 1193–1197 (2006).
8. M. J. Haydon, O. Mielczarek, A. Frank, Á. Román, A. A. R. Webb, Sucrose and ethylene signaling interact to modulate the circadian clock. *Plant Physiol* **175**, 947–958 (2017).
9. S. Balasubramanian, S. Sureshkumar, J. Lempe, D. Weigel, Potent induction of Arabidopsis thaliana flowering by elevated growth temperature. *PLoS Genet* **2**, 0980–0989 (2006).
10. L. Sarid-Krebs, *et al.*, Phosphorylation of CONSTANS and its COP1-dependent degradation during photoperiodic flowering of Arabidopsis. *Plant Journal* **84**, 451–463 (2015).
11. C. C. Monte-Bello, *et al.*, A flexible low cost hydroponic system for assessing plant responses to small molecules in sterile conditions. *Journal of Visualized Experiments* **2018** (2018).
12. S. J. Clough, A. F. Bent, Floral dip: A simplified method for Agrobacterium-mediated transformation of Arabidopsis thaliana. *Plant Journal* **16**, 735–743 (1998).
13. S. J. Harrison, *et al.*, A rapid and robust method of identifying transformed Arabidopsis thaliana seedlings following floral dip transformation. *Plant Methods* **2**, 1–7 (2006).
14. A. Paiano, A. Margiotta, M. De Luca, C. Bucci, Yeast Two-Hybrid Assay to Identify Interacting Proteins. *Curr Protoc Protein Sci* **95**, 1–33 (2019).
15. C. L. Andersen, J. L. Jensen, T. F. Ørntoft, Normalization of real-time quantitative reverse transcription-PCR data: A model-based variance estimation approach to identify genes suited for normalization, applied to bladder and colon cancer data sets. *Cancer Res* **64**, 5245–5250 (2004).
16. K. J. Livak, T. D. Schmittgen, Analysis of relative gene expression data using real-time quantitative PCR and the 2-ΔΔCT method. *Methods* **25**, 402–408 (2001).

17. J. J. Olas, *et al.*, Nitrate acts at the Arabidopsis thaliana shoot apical meristem to regulate flowering time. *New Phytologist* **223**, 814–827 (2019).

18. V. Gramma, V. Wahl, RNA In Situ Hybridization on Plant Tissue Sections: Expression Analysis at Cellular Resolution. *Methods in Molecular Biology* **2686**, 331–350 (2023).

19. J. Schindelin, *et al.*, Fiji: An open-source platform for biological-image analysis. *Nat Methods* **9**, 676–682 (2012).

20. I. a Sparkes, J. Runions, A. Kearns, C. Hawes, Rapid, transient expression of fluorescent fusion proteins in tobacco plants and generation of stably transformed plants. *Nat Protoc* **1**, 2019–2025 (2006).
